# Dual-biomarker-triggered fluorescence probes for differentiating cancer cells and revealing synergistic antioxidant effects under oxidative stress†
†Electronic supplementary information (ESI) available: Experimental details, photophysical data and some fluorescence imaging figures. See DOI: 10.1039/c8sc03781g


**DOI:** 10.1039/c8sc03781g

**Published:** 2019-01-11

**Authors:** Changyu Zhang, Qiang-Zhe Zhang, Kun Zhang, Lu-Yuan Li, Michael D. Pluth, Long Yi, Zhen Xi

**Affiliations:** a State Key Laboratory of Organic-Inorganic Composites , Beijing University of Chemical Technology (BUCT) , 15 Beisanhuan East Road, Chaoyang District , Beijing 100029 , China . Email: yilong@mail.buct.edu.cn; b State Key Laboratory of Medicinal Chemical Biology , College of Pharmacy , Nankai University , Tianjin 300071 , China . Email: liluyuan@nankai.edu.cn; c Materials Science Institute , Institute of Molecular Biology , Department of Chemistry and Biochemistry , University of Oregon , Eugene , OR 97403 , USA; d State Key Laboratory of Elemento-Organic Chemistry , College of Chemistry , National Pesticide Engineering Research Center (Tianjin) , Collaborative Innovation Center of Chemical Science and Engineering , Nankai University , China . Email: zhenxi@nankai.edu.cn

## Abstract

Hydrogen sulfide (H_2_S) and human NAD(P)H:quinine oxidoreductase 1 (hNQO1) are potential cancer biomarkers and also vital participants in cellular redox homeostasis. Simultaneous detection of these two biomarkers would benefit the diagnostic precision of related cancers and could also help to investigate their crosstalk in response to oxidative stress. Despite this importance, fluorescent probes that can be activated by the dual action of H_2_S detection and hNQO1 activity have not been investigated. To this end, dual-biomarker-triggered fluorescent probes **1** and **2** were rationally constructed by installing two chemoselective triggering groups into one fluorophore. Probe **1** provides a small turn-on fluorescence response toward H_2_S but a much larger response to both H_2_S and hNQO1 in tandem. By contrast, fluorescence probe **2** is activated only in the presence of both H_2_S and hNQO1. Probe **2** exhibits a large fluorescence turn-on (>400 fold), high sensitivity, excellent selectivity as well as good biocompatibility, enabling the detection of both endogenous H_2_S and hNQO1 activity in living cells. Bioimaging results indicated that probe **2** could differentiate HT29 and HepG2 cancer cells from HCT116, FHC and HeLa cells owing to the existence of relatively high endogenous levels of both biomarkers. Expanded investigations using **2** revealed that cells could generate more endogenous H_2_S and hNQO1 upon exposure to exogenous hydrogen peroxide (H_2_O_2_), implying the synergistic antioxidant effects under conditions of cellular oxidative stress.

## Abstract 2

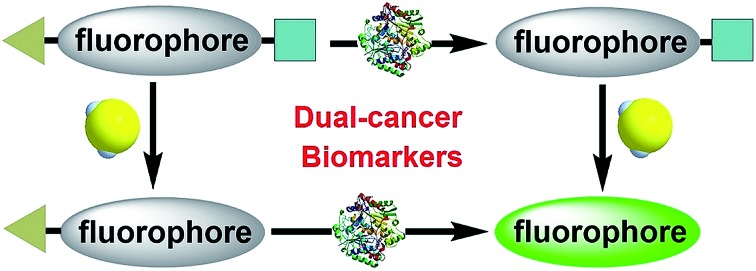
Dual-biomarker-triggered fluorescent probes were developed for simultaneous detection of the two biomarkers H_2_S and hNQO1 in cancer cells.

## Introduction

Cancer, one of the most life-threating diseases, is characterized as uncontrolled growth and division of normal cells beyond their natural boundaries. The mortality of cancer remains high, which is mainly due to metastasis of primary cancer tumors.^1^ The early stages of cancer development carry the maximum potential for therapeutic interventions, and the survival rate of certain cancers can be significantly improved with early diagnosis and treatment.^2^ Cancer biomarkers are endogenous molecules that are differentially expressed in cancer cells relative to their normal counterparts. Altered levels of such biomarkers can be measured to establish a correlation with the disease process and are useful for cancer diagnosis and therapy.^3^ Furthermore, the simultaneous detection of multiple biomarkers can significantly increase diagnostic accuracy.^4^ Recent research has demonstrated that hydrogen sulfide (H_2_S) and human NAD(P)H:quinine oxidoreductase 1 (hNQO1, EC 1.6.99.2) are potential biomarkers in certain cancer biology, which suggests that fluorescent probes that detect these two species simultaneously would be of significant utility.

As the third endogenous gasotransmitter, H_2_S is enzymatically generated from cystathionine γ-lyase (CSE), cystathionine-β-synthase (CBS) and 3-mercaptopyruvate sulfurtransferase (3-MPST)/cysteine aminotransferase (CAT).^5^ H_2_S plays important roles in various biological and pathological progress,^6^ and misregulation of endogenous H_2_S is associated with numerous diseases.^7^ Specially, low levels of endogenous H_2_S appear to exhibit pro-cancer effects, whereas higher concentrations of H_2_S can lead to mitochondrial inhibition and cell death.^8^ We note that some cancer cells, such as ovarian and colorectal cancer cell lines, exhibit increased H_2_S production.^9^ This increased H_2_S may be useful for cell growth and proliferation due to H_2_S-induced angiogenesis.9*c* hNQO1 is a FAD-dependent flavoprotein that catalyzes the obligatory 2-electron reduction of quinones to hydroquinones and provides versatile cytoprotection with multiple functions.^10^ Levels of this reductase are elevated in a number of cancer types, including non-small cell lung cancer, colon cancer, liver cancer and breast cancer,^11^ when compared to the surrounding normal tissue, making it an important cancer biomarker as well as an activator for some anticancer drugs.^12^

In addition to their roles as potential cancer biomarkers, both H_2_S and hNQO1 are also vital participants in cellular redox homeostasis. H_2_S is recognized as a potential antioxidant,^13^ can reduce disulfide bonds, and can react with various reactive oxygen and nitrogen species. For example, Chang *et. al*. reported that vascular endothelial growth factor (VEGF)-triggered H_2_S production is dependent on NADPH oxidase-derived H_2_O_2_.14*a* More recently, we as well as other groups found that endogenous H_2_S can be generated upon simulation of H_2_O_2_ through the glutathionylation and subsequent increased activity of CBS in HEK 293 cells.14b,c In addition, hNQO1 can reduce ubiquinone and vitamin E quinone to their active antioxidant forms and can also reduce superoxide to protect cells during oxidative stress.^15^ Furthermore, hNQO1 can be an intracellular source of NAD^+^, which can fuel the activity of sirtuins to inhibit mitochondrial reactive oxygen production.^16^ Despite the importance of H_2_S and hNQO1 in these systems, the response of these two biomarkers to oxidative stress remains largely unknown. To this end, our goal was to rationally design fluorescent probes for simultaneous detection of H_2_S and hNQO1 to provide new chemical tools for investigating their possible crosstalk in redox homeostasis.

Recent research has demonstrated that fluorescence-based methods are highly suitable and sensitive for *in situ* and real-time visualization of biomolecules.^17^ Numerous fluorescent probes have been developed for the detection of hNQO1 or H_2_S in living systems.^18^ Until now, however, none of these probes allows for the simultaneous detection of the chemical (H_2_S) and enzymatic (hNQO1) biomarkers *via* a single probe. To achieve this goal, we utilized a dual-reactive and dual-quenching strategy, which we reasoned would improve the sensitivity and selectivity of the system.^19^ Dual-activation probes have recently gained attention due to their ability to fine-tune responses by requiring the presence of two specific analytes. For example, Chang *et. al*. reported the dual-analyte detection of H_2_O_2_ and caspase 8 activity during acute inflammation in living mice.20*a* Similar strategies have also been used for the successful dual-analyte detection of small molecules.20b–d Herein, we report the rational design and preparation of H_2_S and hNQO1 dual-responsive fluorescent probes **1** and **2**, which were successfully utilized to differentiate cancer cells and reveal the synergistic antioxidant effects in response to the oxidative stress.

## Results and discussion

### Rational design of the dual-biomarker-triggered fluorescence probes

To enable the simultaneous detection of H_2_S and hNQO1, we installed two chemoselective trigger groups that respond to H_2_S and hNQO1, respectively, into one fluorophore. Such dual-activity probes are superior to traditional single-analyte detection probes because they provide specific advantages, including: (1) avoiding inhomogeneous intracellular distribution from different probes; (2) providing an enhanced off–on response due to the dual-quenching effects; and (3) enable a simple method to investigate the cooperative relationship of the analytes.

**Scheme 1:**
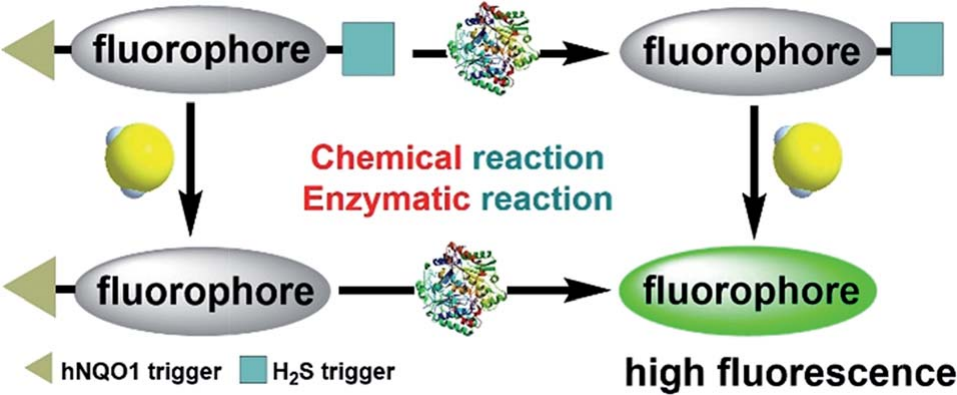
Schematic illustration of the design for a dual-biomarker-triggered fluorescent probe, which should only be activated by the synergistic chemical reaction with H_2_S and enzymatic reaction with hNQO1.

To enable access to such dual-responsive probes, we made use of the trimethyl-lock containing quinone propionic acid (Q_3_PA) moiety reported by McCarley's group18*a* as the triggering group for hNQO1. For the H_2_S detection motif, we utilized the thiolysis of NBD (7-nitro-1,2,3-benzoxadiazole) amines,^21^ which has been utilized by our group as well as others for development of excellent H_2_S probes. Additionally, this H_2_S sensing motif has been used for different biological applications including tumor bioimaging in mice.9*c* Therefore, we combined the Q_3_PA and NBD amine moieties onto coumarin and naphthalimide fluorophores to access dual-responsive systems. The Q_3_PA moiety can switch off the fluorescence of the fluorophore by the photoinduced electron transfer (PET) effect, while the NBD part can quench the fluorescence through the fluorescence resonance energy transfer (FRET) effect. We expected that the fluorescence of the coumarin and naphthalimide fluorophores would be quenched efficiently from this dual-quenching strategy, and that only dual activation of both the Q_3_PA and NBD motifs would result in fluorescence turn-on (Scheme 1).

### Synthesis and optical properties of the probes

**Fig. 1:**
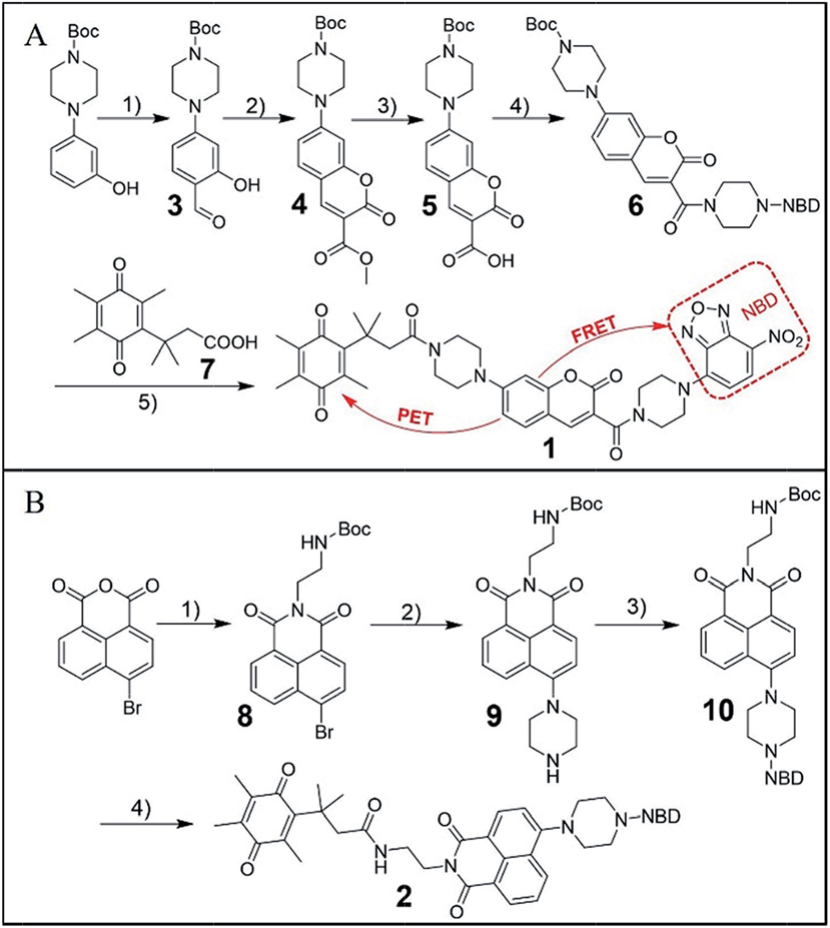
(A) Synthesis route for probe **1**. (1) POCl_3_, DMF, rt, 34%; (2) dimethyl malonate, piperidine, rt, 84%; (3) THF/10% NaOH = 1 : 1, rt, then 1 M HCl, 70%; (4) NBD-PZ, EDCI, DMAP, rt, 67%; (5) DCM/TFA = 1/1, then Q_3_PA, EDCI, DMAP, rt, 44%. (B) Synthesis route for probe **2**. (1) *N*-Boc-ethylenediamine, reflux, 65%; (2) piperazine, reflux, 63%; (3) NBD-Cl, TEA, rt, 66%; (4) DCM/TFA = 1/1, then Q_3_PA, EDCI, DMAP, rt, 88%.

As outlined in Fig. 1A, the synthesis of probe **1** started from a formylation reaction to generate **3**, which was treated with dimethyl malonate to form the coumarin derivative **4**. Then, single-reactive probe **6** was synthesized from coupling 4-nitro-7-piperazinobenzofurazan (NBD-PZ) and the hydrolysis product **5**. After *N*-boc deprotection and further coupling with Q_3_PA, probe **1** was obtained with relative good overall yield. Probe **2** was prepared from a simple four-step synthesis from commercial available reagents (Fig. 1B). 4-Bromo-1,8-naphthalic anhydride was refluxed with *N*-boc-ethylenediamine to produce **8**, after which the piperazinyl group was introduced through a nucleophilic substitution to form **9**. Further reaction with NBD-Cl afforded **10**, which was then deprotected and coupled with the Q_3_PA motif to provide probe **2** in good yield. All compounds were characterized by ^1^H and ^13^C{^1^H} NMR spectroscopy as well as high-resolution mass spectrometry (HRMS) (see ESI†).

**Fig. 2:**
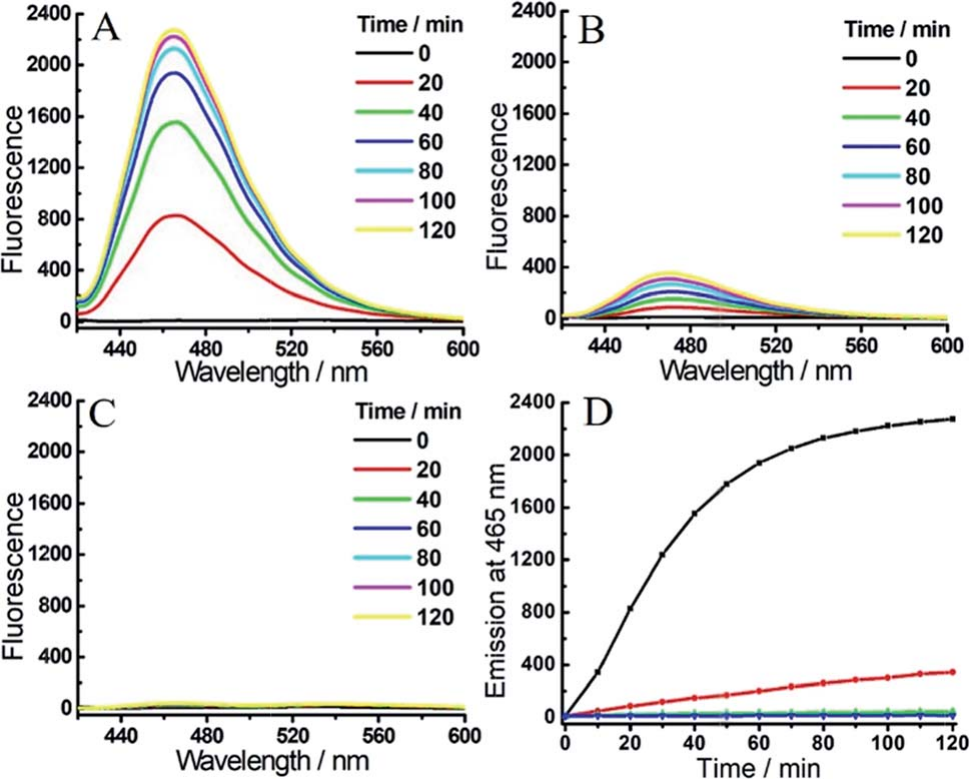
Time-dependent fluorescent response of probe **1** (1 μM) toward H_2_S (200 μM) and/or hNQO1 (1 μg mL^–1^). (A) **1** was treated with H_2_S and hNQO1 simultaneously, or only with H_2_S (B) or hNQO1 (C). (D) Time-dependent emissions at 465 nm for **1** treated with hNQO1 and H_2_S (black), hNQO1 (green), H_2_S (red) or probe **1** alone (blue) in PBS buffer.

With the probes in hand, we examined the optical response of **1** toward H_2_S and hNQO1 in phosphate buffered saline (PBS, 50 mM, pH 7.4 containing 0.007% BSA, 100 μM NADH). As shown in Fig. S1,† **1** displayed two absorption maxima around 405 nm and 500 nm due to the coumarin and NBD amine moieties, respectively. After reaction with both H_2_S and hNQO1, new peaks appeared at 395 and 520 nm, which corresponded to the production of coumarin fluorophore and NBD-SH, respectively.19*b* Notably, **1** remained water-solubile at concentrations over 25 μM (Fig. S2†). Prior to activation, **1** was essentially non-fluorescent (*Φ*_1_ = 0.15%) due to the PET-FRET dual-quenching effect. After treatment with both hNQO1 (1 μg mL^–1^) and H_2_S (200 μM) for 2 h, a large increase in emission (220-fold) appeared at 465 nm (Fig. 2A). When **1** was treated by H_2_S alone for 2 h, only a 34-fold fluorescence enhancement was observed (Fig. 2B), which was far lower than the response from hNQO1 and H_2_S together. When **1** was treated with hNQO1 alone for 2 h, the emission enhancement was negligible (2-fold) (Fig. 2C), implying a more efficient quenching from the NBD moiety in **1**. Stability investigations showed that **1** was stable in PBS buffer in the absence of analytes (Fig. 2D). Taken together, probe **1** can be used to detect H_2_S and hNQO1 in tandem, whereas treatment with only one of the analytes resulted in a significantly smaller response.

**Fig. 3:**
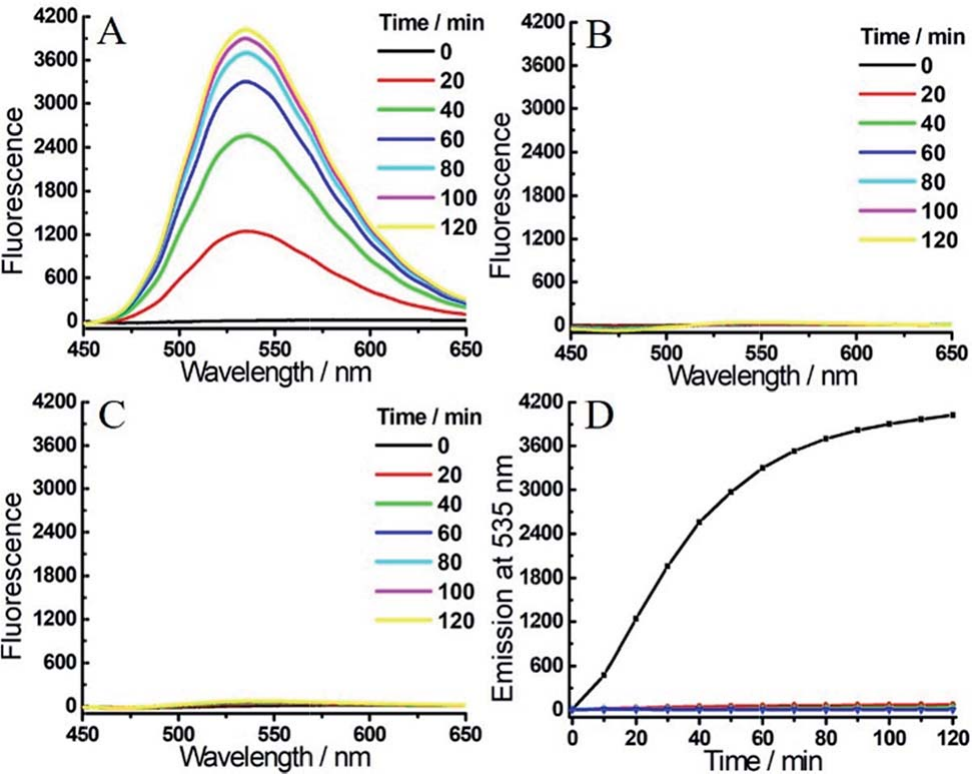
Time-dependent fluorescent response of probe **2** (1 μM) toward H_2_S (200 μM) and/or hNQO1 (1 μg mL^–1^). (A) **2** was treated with H_2_S and hNQO1 simultaneously, or only with hNQO1 (B) or H_2_S (C). (D) Time-dependent emissions at 535 nm for **2** treated with hNQO1 and H_2_S (black), hNQO1 (green), H_2_S (red) or probe **2** alone (blue) in PBS buffer.

To achieve a more efficient single- and dual-quenching effect, we next assessed the fluorescence response of **2** toward H_2_S and/or hNQO1. Emission spectra were also recorded in PBS buffer in the presence of NADH. As shown in Fig. 3A, **2** (*Φ*_2_ = 0.041%) was essentially non-fluorescent due to the dual-quenching effect, but a strong emission at 535 nm was observed when hNQO1 and H_2_S were added simultaneously. After 2 h, the fluorescence increase at 535 nm was over 400-fold. Consistent with our design, treatment of **2** with hNQO1 or H_2_S alone resulted in only a negligible fluorescence enhancement (3- or 7-fold, Fig. 3B–D and S3†). When compared with probe **1**, we found that probe **2** not only resulted in a larger fluorescence turn-on for combined H_2_S/hNQO1 activation, but also exhibited a lower single-analyte response. Because of these positive properties, we utilized probe **2** for subsequent bioimaging investigations.

**Fig. 4:**
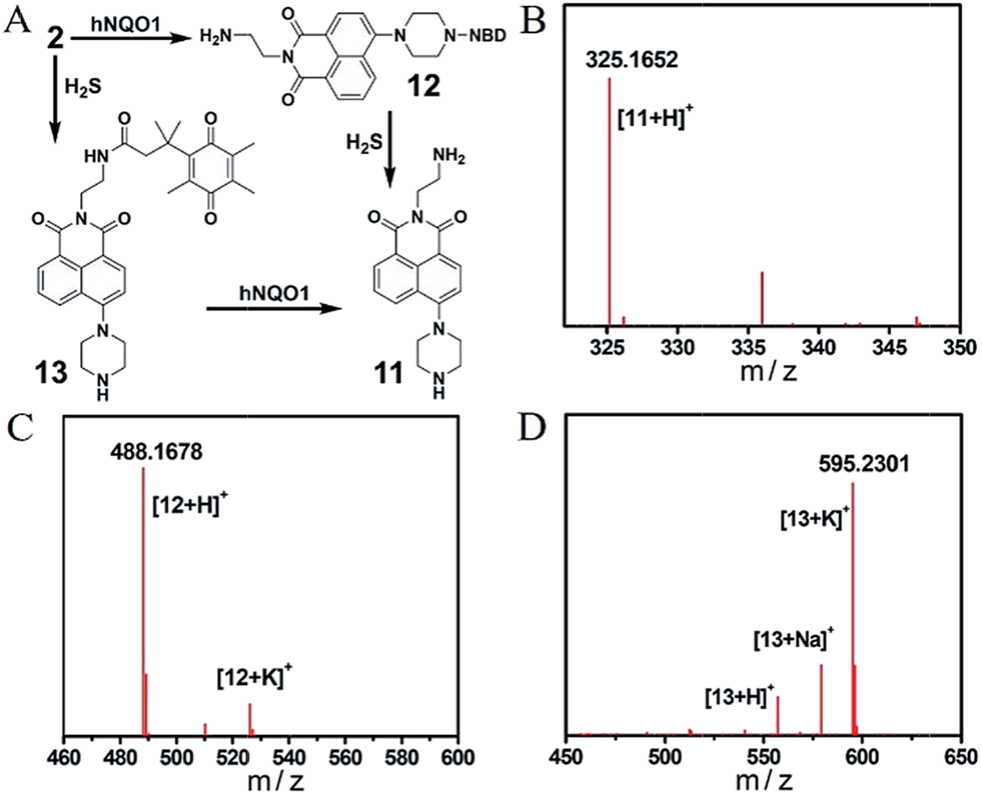
(A) Chemical structures of products from the single or dual reactions of **2** with H_2_S and hNQO1. HRMS spectra of compounds **11** (B), **12** (C) and **13** (D).

Encouraged by the primary fluorescence data, we further validated the chemistry associated with the sensing mechanism by using HRMS and UV-vis analysis. We first confirmed the products of both the single- and dual-analyte reactions of **2** with H_2_S and/or hNQO1 with HRMS (Fig. 4 and S4†). Product **11** (*Φ*_3_ = 7.0%) of the dual activation reaction was observed as [M + H]^+^ 325.1652 (calcd for C_18_H_21_N_4_O_2_^+^, 325.1659). The hNQO1-triggered product **12** and H_2_S-triggered product **13** were observed as [M + H]^+^ 488.1678 (calcd for C_24_H_22_N_7_O_5_^+^, 488.1677) and [M + K]^+^ 595.2301 (calcd for C_32_H_36_KN_4_O_5_^+^, 595.2317), respectively. We did not observe the cross reaction side-products (*e.g.* hNQO1-triggered **13** or H_2_S-triggered **12**) in the MS spectra. We next performed UV-vis experiments to further probe the reaction mechanism. As shown in Fig. S5A,† the absorption spectrum of **2** displayed two maximum absorbance peaks near 350 and 500 nm. After treatment with H_2_S and hNQO1, both of these peaks disappeared and were replaced by peaks at 400 and 520 nm, which corresponded to the fluorophore and NBD-SH, respectively. When H_2_S alone was added, new peaks at 400 and 520 nm were also observed (Fig. S5B†). Furthermore, there was an obvious overlap between the absorbance profile of NBD-PZ and the emission profile of **11**, indicating an intramolecular FRET effect in probe **2** (Fig. S5C†). When **2** was treated by hNQO1 alone, the absorbance peak at 500 nm increased (Fig. S5D†), implying that the PET process was abolished because the PET effect should result in small changes in absorbance spectra.^22^ In addition, probe **2** maintained water solubility of over 20 μM under the experimental conditions (Fig. S6†).

**Fig. 5:**
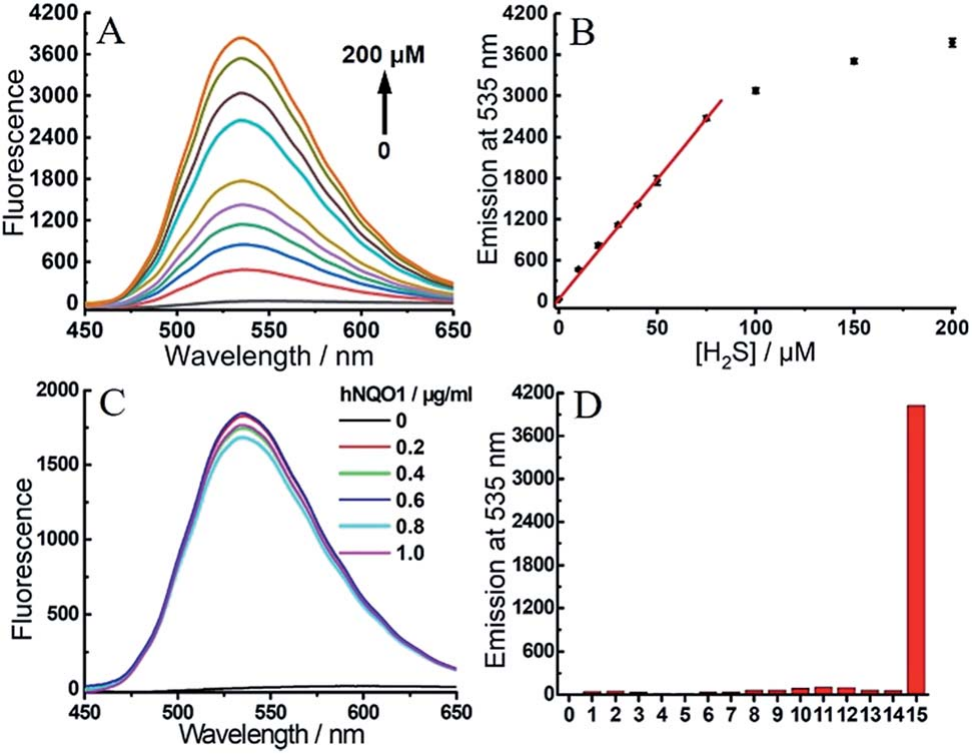
(A) Emission spectra of **2** (1 μM) toward different concentrations of H_2_S (0–200 μM) in the presence of hNQO1 (1 μg mL^–1^). (B) Linear relationship (*R*^2^ = 0.999 up to 75 μM) between the emission at 535 nm from **2** and the concentration of H_2_S. (C) Emission spectra of **2** (1 μM) toward different levels of hNQO1 (0–1 μg mL^–1^) in the presence of H_2_S (50 μM). (D) Emissions at 535 nm of **2** (1 μM) after treatment with various biologically-relevant species. Lane 0, probe **2** alone; lanes 1–7, SO_3_^2–^ (200 μM), S_2_O_3_^2–^ (200 μM), Cys (500 μM), Hcy (500 μM), GSH (5 mM), H_2_O_2_ (200 μM), HClO (200 μM), respectively, all in the presence of H_2_S (200 μM); lanes 8–14, SO_3_^2–^ (200 μM), S_2_O_3_^2–^ (200 μM), Cys (500 μM), Hcy (500 μM), GSH (5 mM), H_2_O_2_ (200 μM), HClO (200 μM), respectively, all in the presence of hNQO1 (1 μg mL^–1^); lane 15, H_2_S (200 μM) and hNQO1 (1 μg mL^–1^).

To gain more detailed information about the sensitivity of the dual-responsive probe, we incubated **2** with different levels of hNQO1 and H_2_S for 2 h, after which the emission profiles were measured. Probe **2** was first treated with different concentrations of H_2_S (0–200 μM) in the presence of hNQO1 (1 μg mL^–1^). As shown in Fig. 5A and B, the emission at 535 nm was linearly related to the concentrations of H_2_S from 0 to 75 μM. When added to 1 μg mL^–1^ hNQO1, a 10 μM H_2_S solution resulted in a 46-fold fluorescence response. Similarly, we treated **2** with various levels of hNQO1 (0.2–1 μg mL^–1^) in the presence of a constant H_2_S concentration (50 μM), and observed a fluorescence enhancement of 180-fold (Fig. 5C).

One major requirement for a fluorescent probe is that it must exhibit a response toward the targeted analytes but not for other competing species. In order to confirm that the turn-on response of **2** was selectively caused by the dual activation of hNQO1 and H_2_S, probe **2** was incubated with different reactive sulfur species (SO_3_^2–^ and S_2_O_3_^2–^), biothiols (Cys, Hcy and GSH) and reactive oxygen species (H_2_O_2_ and HClO) in the presence of hNQO1 or H_2_S. As shown in Fig. 5D, only the co-incubation of hNQO1 and biothiols could trigger a very slight fluorescence response (<10-fold, lanes 10–12), which was significantly lower than the response triggered by hNQO1 and H_2_S (>400-fold, lane 15). No fluorescence increase was observed when H_2_O_2_ or HClO was added (lanes 6–7 and 13–14). Furthermore, treatment of 2 with dicoumarol, an hNQO1 inhibitor, resulted in a slower reaction rate than the inhibitor-free controls, confirming the requirement of hNQO1 for probe activation (Fig. S7†).

### Differentiation of cancer cells using the probe **2**

We first evaluated the cytotoxicity of **2** in HT29 cells (human colorectal epithelial cancer cells) by using the methyl thiazolyl tetrazolium (MTT) assay. The results showed that after 2 h of cellular internalization of 33 μM probe, more than 90% of the cells remained viable (Fig. S8†), implying a low cytotoxicity of **2**. The cytotoxicity of **2** was further studied in HEK293A cells (human embryonic kidney cells) by monitoring of adherent cell proliferation through the xCELLigence RTCA system (Fig. S9†). Compound **2** did not show significant cytotoxicity from 0–15 μM after 24 h incubation, and therefore 10 μM of **2** was used for bioimaging experiments. To investigate whether **2** could be employed to distinguish different types of cancer cells, several cell types were chosen as model biological systems. Given the elevated levels of both H_2_S and hNQO1 in some colorectal cancer cells, HT29 and HCT116 cells (human colorectal epithelial cancer cell lines) as well as FHC cells (human normal colorectal epithelial cell line) were initially selected.9*c* Then HepG2 cells (human liver cancer cells) with a high level of endogenous H_2_S and HeLa cells (human cervical cancer cells) with a low level of endogenous H_2_S were also introduced.^8^

**Fig. 6:**
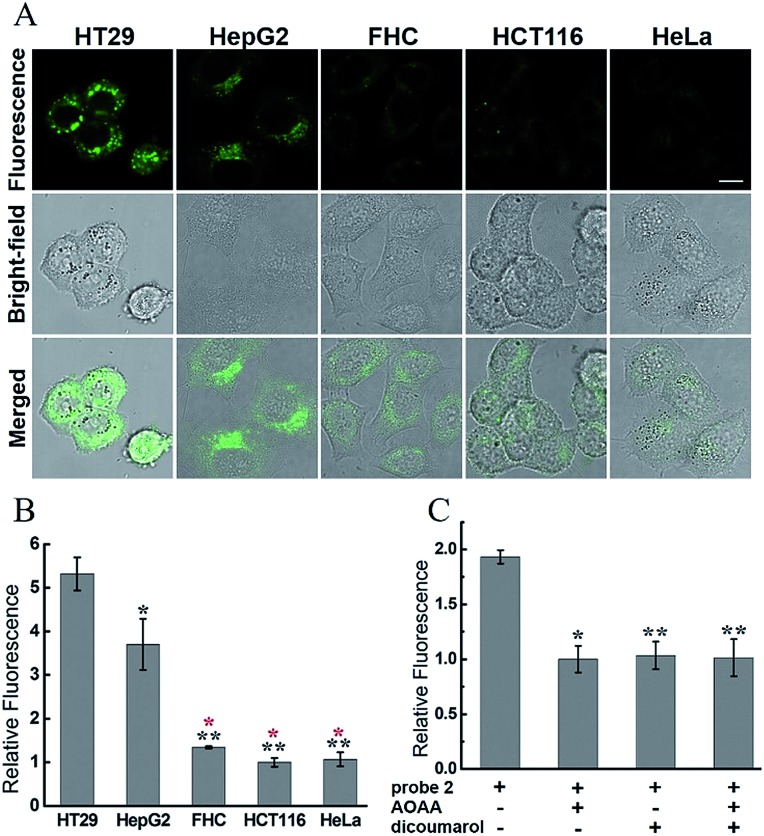
Confocal microscopy images for endogenous H_2_S and hNQO1 detection in living cells using **2**. (A) Cells (∼2 × 10^4^ cells per well) were incubated with only **2** (10 μM) for 1 h, washed, then imaged. Scale bar, 10 μm. (B) Relative fluorescence intensity of images from (A). (C) Relative fluorescence intensity of images from inhibitor-pretreated HT29 cells. *N* = 3 fields of cells, error bars are means ± sd. *P < 0.05; **P < 0.01. For (B), the black * was relative to HT29 group, and the red * was relative to HepG2 group.

We assumed that only the **2**-stained cells with relatively high endogenous levels of both H_2_S and hNQO1 would display significant fluorescence. Aligned with this expectation, the confocal fluorescence images showed clearly differentiable responses from the selected cells (Fig. 6A). The fluorescence intensity in HT29 and HepG2 cells was much stronger than that in other cell lines. The relative fluorescence increases in HT29 and HepG2 cells were about 5.3 and 3.7 fold higher than that of other cells (Fig. 6B). The significantly different fluorescence observed in cancerous *versus* non-cancerous cells is consistent with the probe design and suggests that the probe is differentially activated in cancerous *versus* non-cancerous cells.

In control experiments for single biomarker detection, two single-analyte probes **NIR-H_2_S** (for H_2_S detection)9*c* and **NIR-hNQO1** (for hNQO1 detection)^23^ developed by us were separately incubated with these cells (Fig. S10†). As shown in Fig. S11,† when cells were treated with **NIR-H_2_S**, the HT29, HepG2 and HCT116 cells displayed a fluorescence response, implying the existence of endogenous H_2_S in the cells. When cells were incubated with **NIR-hNQO1**, the observed fluorescence from the HT29 and HepG2 cells was stronger than that from the other three cell lines (Fig. S12†). The results indicated the relatively high endogenous levels of both H_2_S and hNQO1 in HT29 and HepG2 cells, which is consistent with the bioimaging results of probe **2**.

To further confirm the dual-activation of **2** in cancer cells, we added aminooxyacetic acid (AOAA, 200 μM), which is an inhibitor of enzymatic H_2_S synthesis, and dicoumarol (100 μM), which is an hNQO1 inhibitor. For the inhibitor-treated groups, HT29 cells were pretreated with the inhibitor for 30 min, then incubated with **2** (10 μM) for 1 h, washed and imaged (Fig. S13†). HT29 cells showed strong fluorescence after incubation with **2** alone for 1 h. In contrast, pretreatment of one or two inhibitors led to a significant decrease in fluorescence, and the observed fluorescence intensity was about a half of that in the group without inhibitors (Fig. 6C). These results clearly demonstrated the dual H_2_S and hNQO1 requirement for **2**.

### Investigation of the crosstalk between H_2_S and hNQO1 under oxidative stress

**Fig. 7:**
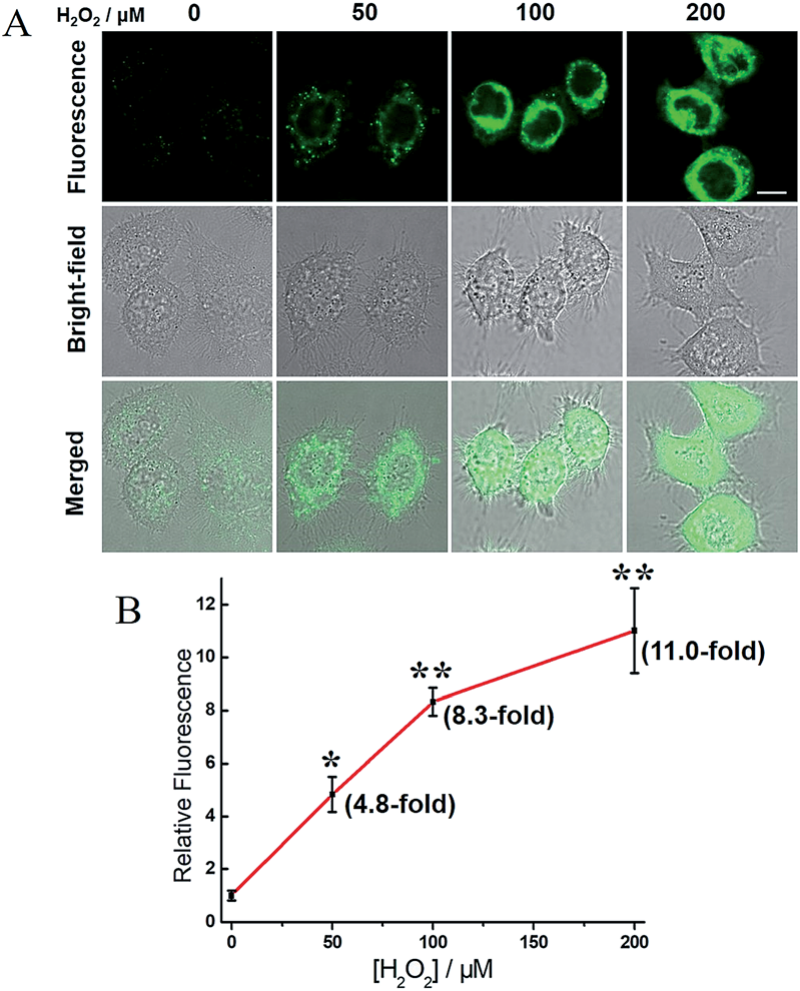
Confocal microscopy images for concentration-dependent H_2_O_2_-induced fluorescence in living HeLa cells using **2**. (A) Cells were co-incubated with probe **2** (10 μM) and H_2_O_2_ (0, 50, 100 or 200 μM) for 1 h, washed and imaged. Scale bar, 10 μm. (B) Relative fluorescence intensity of images *versus* H_2_O_2_ concentration. *N* = 3 fields of cells, error bars are means ± sd. *P < 0.05; **P < 0.01.

**Fig. 8:**
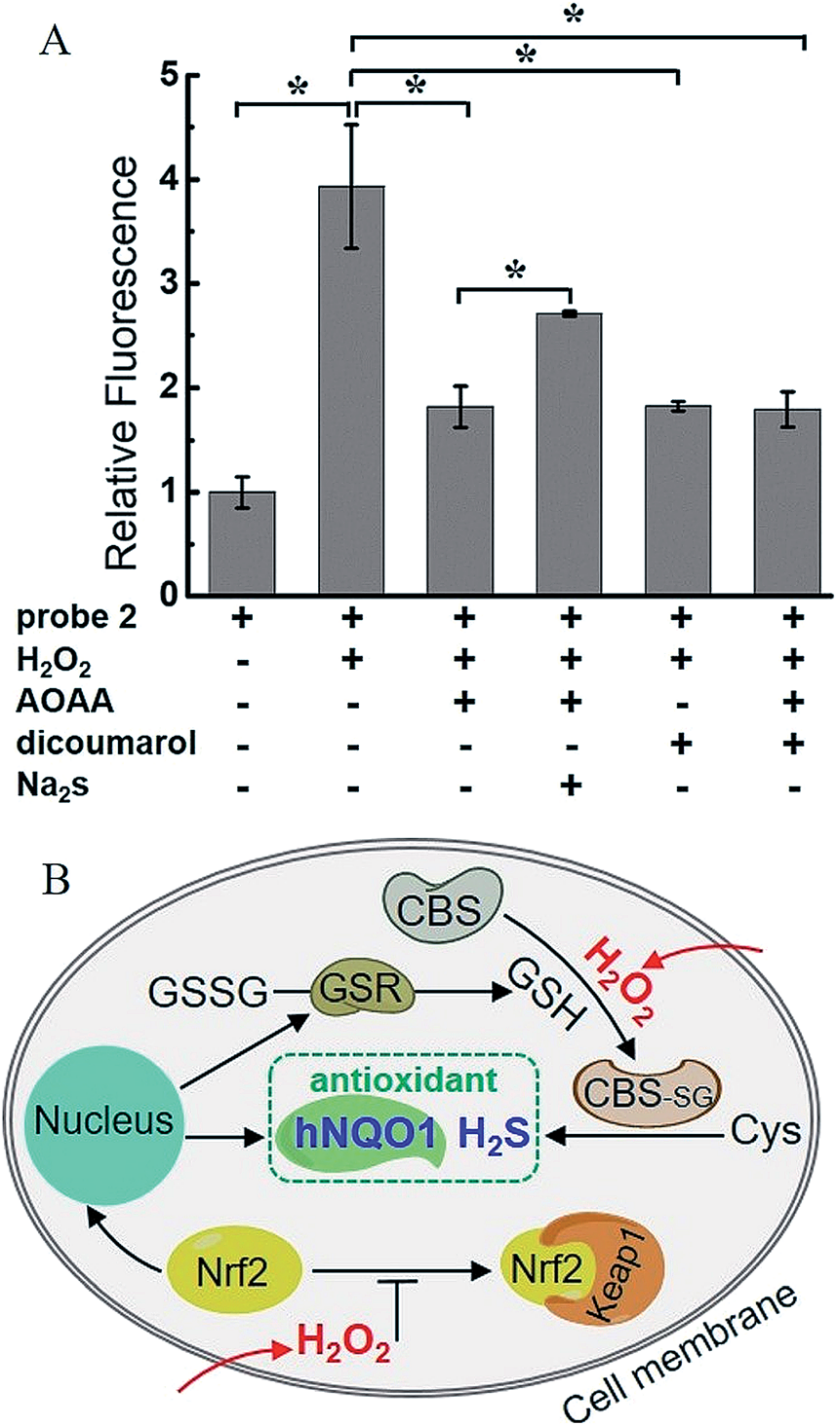
(A) Relative fluorescence intensity of confocal microscopy images from H_2_O_2_-induced HeLa cells. *N* = 3 fields of cells, error bars are means ± sd. *P < 0.05. (B) A proposed mechanism of the synergistic antioxidant effect of H_2_S and hNQO1 under oxidative stress.

H_2_O_2_, a common ROS, was introduced as a stimulus to investigate the potential crosstalk between H_2_S and hNQO1 in cellular redox homeostasis. HeLa cells were selected as the model biological systems due to the relative low levels of the both endogenous biomarkers. The cells were stained by **2**, washed and imaged. As displayed in Fig. 7, **2**-stained HeLa cells exhibited very weak fluorescence. However, a significant fluorescence response was observed when cells were co-incubated with **2** and H_2_O_2_ (50, 100 or 200 μM) for 1 h. To further understand the results, the inhibitors AOAA and dicoumarol were also used for control experiments (Fig. S14†). The H_2_O_2_-stimulated cells displayed a significant fluorescence decrease when pretreated with one or both inhibitors. The relative emission (Fig. 8A) showed that the stimulation by H_2_O_2_ could trigger about 3.9-fold fluorescence enhancement, which was much higher than the inhibitor-pretreated control groups (about 1.8-fold). In addition, after co-incubation with H_2_O_2_ and **2**, AOAA-pretreated cells were further treated with Na_2_S (150 μM) for 30 min, and a small increase in fluorescence was observed (1.5 fold) when compared with the AOAA-pretreated control group. These data suggest that endogenous H_2_S and hNQO1 could be spontaneously generated in living cells when cells were suffering from acute oxidative stress caused by exogenous H_2_O_2_.

Based on current knowledge, hNQO1 is regulated by the Keap1 (Kelch-like ECH-associated protein 1)/Nrf2 (nuclear factor-erythroid 2-related factor 2)/ARE (antioxidant response elements) pathway.^10^ Nrf2 protein levels can rapidly increase in response to ROS, triggering the expression of hNQO1 to inhibit the formation of free radicals.15a,^24^ Meanwhile, elevated Nrf2 can increase the expression of glutathione reductase (GSR), which can reduce GSSG to GSH.24d,e Such GSH can be involved in the S-glutathionylation of CBS under H_2_O_2_ to produce CBS_-SG_, which would enable more efficient biosynthesis of endogenous H_2_S.14b,c Thus, we propose that the synergistic antioxidant effect of H_2_S and hNQO1 for handling oxidative stress in living cells is possibly regulated by Nrf2, which can trigger the expression of hNQO1 directly and improve endogenous H_2_S levels indirectly through controlling GSH (Fig. 8B). Taken together, these results support a synergistic antioxidant effect under cellular oxidative stress.

## Conclusions

In summary, dual-biomarker-triggered fluorescent probes were developed for the simultaneous detection of two potential cancer biomarkers. Probe **1** could detect the two biomarkers with a slight fluorescence response toward one biomarker (34-fold turn-on) and a significantly enhanced fluorescence by dual activation (220-fold turn-on). By contrast, the fluorescence of probe **2** was significantly enhanced and showed a greater response for the dual-activation from H_2_S and hNQO1 (>400-fold turn-on). Moreover, probe **2** exhibited high sensitivity, excellent selectivity and good biocompatibility, which enabled us to differentiate activation levels in HT29 and HepG2 cells from FHC, HCT116 and HeLa cells due to the notably different endogenous levels of H_2_S and hNQO1 in the cell lines. Importantly, using the probe **2**, we revealed a synergistic antioxidant effect between H_2_S and hNQO1 in living cells in response to the oxidative stress. These results clearly demonstrate the strengths of this dual reporter system, including the significant off–on response, ability to distinguish cancer cells with both cancer biomarkers, and ability to investigate the crosstalk of analytes. We also note, however, potential limitations of this system. For example, the developed tools only provide information on the relative levels of the biomarkers in different cell lines rather than precise quantification measurements. In addition, the development of probes with longer wavelength emissions would be needed to translate these systems into more complex systems, such as animal studies. Based on these needs, we are currently working to develop related dual-responsive probes with emission in the near-infrared region for *in vivo* applications. Overall, our work has demonstrated the research potential of dual-responsive fluorescent probes in cancer biology and intracellular redox homeostasis.

## Conflicts of interest

The authors declare no competing financial interests.

## Supplementary Material

Supplementary information
