# Supplementary material for: Dual-biomarker-triggered fluorescence probes for differentiating cancer cells and revealing synergistic antioxidant effects under oxidative stress
Source: Chem Sci. 2019 Jan 11;10(7):1945–52. doi: 10.1039/c8sc03781g (PMC6399676; doi:10.1039/c8sc03781g)
Supplement: Supplementary file 1 [file SC-010-C8SC03781G-s001.pdf]

Electronic Supplementary Material (ESI) for Chemical Science.
This journal is © The Royal Society of Chemistry 2019

Supporting Information

Dual-biomarker-triggered fluorescence probes for differentiating cancer cells and revealing synergistic antioxidant effects under oxidative stress

Changyu Zhang,[‡a] Qiang-Zhe Zhang,[‡b] Kun Zhang,[‡b] Lu-Yuan Li,*[b] Michael D. Pluth,[c] Long Yi*[a] and Zhen Xi*[d]

[a]*State Key Laboratory of Organic-Inorganic Composites, Beijing University of Chemical Technology (BUCT), 15 Beisanhuan East Road, Chaoyang District, Beijing 100029, China. E-mail: yilong@mail.buct.edu.cn*

[b]*State Key Laboratory of Medicinal Chemical Biology and College of Pharmacy, Nankai University, Tianjin 300071, China. E-mail: liluyuan@nankai.edu.cn*

[c]*Materials Science Institute, Institute of Molecular Biology, Department of Chemistry and Biochemistry, University of Oregon, Eugene, OR 97403, USA*

[d]*State Key Laboratory of Elemento-Organic Chemistry, College of Chemistry, National Pesticide Engineering Research Center (Tianjin), Collaborative Innovation Center of Chemical Science and Engineering, Nankai University, China. E-mail: zhenxi@nankai.edu.cn*

[‡]These authors contributed equally to this work.

Table of contents

General chemicals and instruments 3

Synthesis of the probes 3

Spectroscopic studies 7

Cell culture 8

Cytotoxicity assay 8

Confocal fluorescence imaging for living cells 9

References 10

Figure S1 Time-dependent UV-vis spectra of **1** towards H_2S and hNQO1 10

Figure S2 Solubility evaluation of **1** 11

Figure S3 Time-dependent fluorescent response of **2** toward H_2S or hNQO1 11

Figure S4 HRMS spectra of the products of **2** with H_2S and/or hNQO1 12

Figure S5 Time-dependent UV-vis spectra of **2** toward H_2S and/or hNQO1 13

Figure S6 Solubility evaluation of **2** 13

Table 1 Optical properties of **1**, **2** and **11** 13

Figure S7 Fluorescent enzymatic assay of **2** with dicoumarol 14

Figure S8 MTT assay of **2** 14

Figure S9 Study on cytotoxicity of **2** through xCELLigence RTCA system 15

Figure S10 Chemical structures of **NIR-H_2S** and **NIR-hNQO1** 15

Figure S11 Confocal microscopy images of **NIR-H_2S** 16

Figure S12 Confocal microscopy images of **NIR-hNQO1** 16

Figure S13 Confocal microscopy images for inhibitor-treated HT29 cells using **2** 17

Figure S14 Confocal microscopy images for H_2O_2-treated HeLa cells using **2** 17

NMR Spectra 18

HRMS Spectra 27

General chemicals and instruments

All chemicals and solvents used for synthesis were purchased from commercial suppliers and applied directly in the experiments without further purification. The progress of the reaction was monitored by TLC on pre-coated silica plates (Merck 60F-254, 250 μm in thickness), and spots were visualized by UV light. Merck silica gel 60 (70 - 200 mesh) was used for general column chromatography purification. ^{1}H NMR and ^{13}C{^{1}H} NMR spectra were recorded on a Bruker 400 (400 MHz ^{1}H and 101 MHz ^{13}C) spectrometer at room temperature. Chemical shifts are reported in parts per million relative to internal residual solvent peaks ($CDCl_3$ = 7.26 ppm; MeOD = 4.87 ppm; DMSO-d_6 = 2.50 ppm). ^{1}H NMR coupling constants (J) are reported in Hertz (Hz), and multiplicity is indicated as the following: s (singlet), d (doublet), dd (doublet doublet), t (triplet), m (multiplet). High-resolution mass spectra (HRMS) were recorded on an Agilent 6540 UHD Accurate-Mass Q-TOFLC/MS. The UV-visible spectra were obtained on a UV-3600 UV-VIS-NIR spectrophotometer (SHIMADZU, Japan). Fluorescence studies were performed using F-280 spectrophotometer (Tianjin Gangdong Sci & Tech., Development. Co., Ltd). The cellular bioimaging was carried out on a confocal microscope (Leica TCS SP8).

Synthesis of the probes

*Synthesis of **3***. N-Boc-3-piperazin-1-yl-phenol (2.2 g, 8.0 mmol) was dissolved in DMF (5.0 mL) and cooled to 0 °C. Separately, phosphoryl chloride (0.93 mL, 10 mmol) was added to another portion of DMF (5.0 mL) and stirred for 15 min, which was added to the chilled solution dropwise. The reaction mixture was warmed to room temperature and stirred for 5 h before being poured into ice water (100 mL). After adjusting the pH to 8.0 using saturated aqueous $NaHCO_3$, the aqueous phase was extracted with ethyl acetate (50 mL × 2). The combined organic layers were dried by anhydrous Na_2SO_4 and evaporated under reduced pressure. The residue was purified by silica gel column chromatography with CH_2Cl_2 to give an off-white solid **3** (840 mg, 34%). ^{1}H NMR (400 MHz, $CDCl_3$) δ 11.48 (s, 1H), 9.58 (s, 1H), 7.34 (d, J = 8.8 Hz, 1H), 6.44 (dd, J = 8.8, 2.4 Hz, 1H), 6.26 (d, J = 2.3 Hz, 1H), 3.59 - 3.55 (m, 4H),

3.43 - 3.39 (m, 4H), 1.48 (s, 9H).

*Synthesis of **4***. To a solution of **3** (480 mg, 1.6 mmol) and dimethyl malonate (0.31 mL, 2.0 mmol) in methanol (20 mL), piperidine (0.20 mL) was added dropwise. The mixture was stirred at room temperature for 2 h and the solvent was removed under reduced pressure. The residue was purified by silica gel column chromatography with CH_2Cl_2/CH_3OH (100/1) to give a yellow solid **4** (512 mg, 84%). ^1H NMR (400 MHz, $CDCl_3$) δ 8.46 (s, 1H), 7.41 (d, J = 8.9 Hz, 1H), 6.79 (dd, J = 8.9, 2.4 Hz, 1H), 6.62 (d, J = 2.3 Hz, 1H), 3.91 (s, 3H), 3.61 - 3.58 (m, 4H), 3.44 - 3.40 (m, 4H), 1.48 (s, 9H); $^{13}C\{^1H\}$ NMR (101 MHz, $CDCl_3$) δ 164.7, 158.1, 157.9, 155.2, 154.7, 149.5, 131.0, 111.6, 111.3, 109.6, 99.7, 80.6, 52.7, 46.9, 28.5. HRMS (ESI): m/z 389.1705 $[M+H]^+$ (calcd for $C_{20}H_{25}N_2O_6^+$, 389.1707).

*Synthesis of **5***. To a mixed solution of THF (6.0 mL) and 10% aqueous NaOH (6.0 mL), **4** (116 mg, 0.30 mmol) was added, and the mixture was stirred for 40 min at room temperature. The pH of the reaction solution was adjusted to about 3 with 1 M aqueous HCl while cooling in an ice bath to give a yellow precipitate, which was extracted with ethyl acetate (30 mL × 2). Subsequently, the organic phase was washed with brine, dried with anhydrous Na_2SO_4 and concentrated under reduced pressure to afford **5** as a yellow solid (80 mg, 70%). ^1H NMR (400 MHz, DMSO-d_6) δ 8.60 (s, 1H), 7.68 (d, J = 9.0 Hz, 1H), 7.00 (dd, J = 9.0, 2.3 Hz, 1H), 6.82 (d, J = 2.1 Hz, 1H), 3.50 - 3.43 (m, 8H), 1.42 (s, 9H); $^{13}C\{^1H\}$ NMR (101 MHz, DMSO-d_6) δ 164.4, 158.6, 157.4, 154.8, 153.8, 149.2, 131.5, 111.5, 109.9, 108.8, 98.5, 79.2, 46.1, 28.1. HRMS (ESI): m/z 375.1551 $[M+H]^+$ (calcd for $C_{19}H_{23}N_2O_6^+$, 375.1548).

*Synthesis of **6***. To a solution of **5** (44 mg, 0.12 mmol) in CH_2Cl_2 (10 mL), 1-ethyl-3-(3-dimethylaminopropyl)carbodiimide hydrochloride (EDCI, 58 mg, 0.30 mmol) and 4-dimethylaminopyridine (DMAP, 73 mg, 0.60 mmol) were added. After stirred for 5 min, NBD-PZ (37 mg, 0.15 mmol) was added and the mixture was stirred for 2 h at room temperature before solvent evaporation. The crude residue was purified by silica gel column chromatography with CH_2Cl_2/CH_3OH (100/1) to give a red solid **6** (48 mg, 67%). ^1H NMR (400 MHz, DMSO-d_6) δ 8.50 (d, J = 9.1 Hz, 1H), 8.10 (s, 1H), 7.57 (d, J = 8.9 Hz, 1H), 7.00 (dd, J = 8.9, 2.3 Hz, 1H), 6.86 (d, J = 2.2

Hz, 1H), 6.62 (d, *J* = 9.2 Hz, 1H), 4.26 (s, 2H), 4.15 (s, 2H), 3.87 (s, 2H), 3.72 (s, 2H), 3.50 - 3.39 (m, 8H), 1.43 (s, 9H); $^{13}C\{^1H\}$ NMR (101 MHz, DMSO-d_6) δ 164.2, 158.3, 156.1, 153.8, 153.8, 145.4, 144.8, 144.7, 144.1, 136.3, 130.0, 121.4, 117.7, 111.6, 109.2, 103.4, 99.5, 79.2, 46.4, 38.2, 28.1. HRMS (ESI): m/z 606.2293 $[M+H]^+$ (calcd for $C_{29}H_{32}N_7O_8^+$, 606.2307).

Synthesis of probe ***1***. Compound **7** was prepared according our previous work.[1] Compound **6** (33 mg, 0.055 mmol) was dissolved in a mixed solution (3.0 mL, CH_2Cl_2/TFA = 1/1) and stirred for 30 min at room temperature. After solvent evaporation, the residue was dissolved in anhydrous CH_2Cl_2 (10 mL), **7** (15 mg, 0.06 mmol), EDCI (23 mg, 0.12 mmol) and DMAP (30 mg, 0.24 mmol) were added. After stirred overnight, solvent was removed and the residue was subjected to silica gel column chromatography with CH_2Cl_2/CH_3OH (100/1) to get a red solid **1** (18 mg, 44%). ^1H NMR (400 MHz, $CDCl_3$) δ 8.44 (d, *J* = 8.8 Hz, 1H), 8.00 (s, 1H), 7.42 (d, *J* = 8.8 Hz, 1H), 6.83 (dd, *J* = 8.8, 2.3 Hz, 1H), 6.68 (d, *J* = 2.0 Hz, 1H), 6.34 (d, *J* = 8.9 Hz, 1H), 4.28 - 4.19 (m, 4H), 4.07 - 4.00 (m, 2H), 3.75 - 3.68 (m, 6H), 3.53 - 3.46 (m, 2H), 3.42 - 3.36 (m, 2H), 3.06 (s, 2H), 2.16 (s, 3H), 1.95 (s, 3H), 1.91 (s, 3H), 1.47 (s, 6H); $^{13}C\{^1H\}$ NMR (101 MHz, $CDCl_3$) δ 191.6, 187.7, 171.0, 165.0, 158.9, 157.0, 154.3, 154.1, 146.0, 145.0, 144.8, 143.1, 138.5, 137.0, 135.0, 130.1, 124.7, 118.2, 112.0, 110.1, 103.1, 100.3, 49.5, 48.6, 47.2, 46.5, 44.8, 42.1, 40.8, 37.9, 29.8, 29.0, 14.4, 12.8, 12.3. HRMS (ESI): m/z 738.2868 $[M+H]^+$ (calcd for $C_{38}H_{40}N_7O_9^+$, 738.2882).

Synthesis of ***8***. To a solution of 4-bromo-1,8-naphthalic anhydride (443 mg, 1.6 mmol) in EtOH (15 mL), N-boc-ethylenediamine (285 mg, 1.6 mmol) was added. The mixture was heated to reflux for 2 h, then cooled to room temperature to form a white precipitate, which was filtrated and washed with cold EtOH (10 mL), yielding **8** as a white powder (435 mg, 65%). ^1H NMR (400 MHz, $CDCl_3$) δ 8.56 (d, *J* = 7.2 Hz, 1H), 8.44 (d, *J* = 8.3 Hz, 1H), 8.31 (d, *J* = 7.8 Hz, 1H), 7.94 (d, *J* = 7.8 Hz, 1H), 7.76 (t, *J* = 7.8 Hz, 1H), 5.06 (s, 1H), 4.30 (t, *J* = 5.2 Hz, 2H), 3.50 (s, 2H), 1.26 (s, 9H); $^{13}C\{^1H\}$ NMR (101 MHz, $CDCl_3$) δ 163.9, 156.2, 133.3, 132.2, 131.4, 131.1, 130.5, 130.4, 128.9, 128.1, 122.9, 122.0, 79.2, 40.1, 39.5, 28.3. HRMS (ESI): m/z 319.0085

[M-Boc+H]$^+$ (calcd for $C_{14}H_{12}BrN_2O_2^+$, 319.0077).

*Synthesis of **9***. Compound **8** (418 mg, 1.0 mmol) and piperazine (860 mg, 10 mmol) were dissolved in 2-methoxyethanol (20 mL) and heated to reflux for 8 h. After the solvent was removed under reduced pressure, the residue was purified by silica gel column chromatography with CH_2Cl_2/CH_3OH (100/8) to give a yellow solid **9** (267 mg, 63%). ^{1}H NMR (400 MHz, MeOD) δ 8.50 - 8.40 (m, 3H), 7.73 (t, J = 7.9 Hz, 1H), 7.32 (d, J = 8.1 Hz, 1H), 4.24 (t, J = 5.7 Hz, 2H), 3.42 (t, J = 5.7 Hz, 2H), 3.32 - 3.27 (m, 8H), 1.24 (s, 9H); ^{13}C{^{1}H} NMR (101 MHz, MeOD) δ 166.0, 165.6, 158.5, 157.3, 133.5, 132.1, 131.6, 131.1, 127.4, 127.0, 124.3, 118.0, 116.3, 79.9, 53.9, 46.2, 40.9, 39.5, 28.6. HRMS (ESI): m/z 425.2182 [M+H]$^+$ (calcd for $C_{23}H_{29}N_4O_4^+$, 425.2183).

*Synthesis of **10***. To a solution of compound **9** (114 mg, 0.27 mmol) and NBD-Cl (179 mg, 0.90 mmol) in CH_2Cl_2 (15 mL), triethylamine (TEA, 125 μL) was added dropwise. The mixture was stirred for 3 h at room temperature. After the solvent was removed, the resulted residue was subjected to silica gel column chromatography with CH_2Cl_2/CH_3OH (100/1) to give a red solid **10** (105 mg, 66%). ^{1}H NMR (400 MHz, DMSO-d_6) δ 8.60 (d, J = 8.4 Hz, 1H), 8.54 (d, J = 9.1 Hz, 1H), 8.48 (d, J = 7.1 Hz, 1H), 8.40 (d, J = 7.9 Hz, 1H), 7.85 (t, J = 7.8 Hz, 1H), 7.40 (d, J = 8.1 Hz, 1H), 6.87 (t, J = 5.8 Hz, 1H), 6.76 (d, J = 9.2 Hz, 1H), 4.46 (s, 4H), 4.12 (t, J = 6.1 Hz, 2H), 3.57 - 3.50 (m, 4H), 3.28 - 3.20 (m, 2H), 1.22 (s, 9H); ^{13}C{^{1}H} NMR (101 MHz, DMSO-d_6) δ 163.7, 163.2, 155.7, 154.4, 145.4, 144.9, 144.8, 136.3, 131.9, 130.6, 130.4, 129.3, 126.1, 125.2, 122.9, 121.6, 116.5, 115.2, 103.8, 77.4, 51.9, 49.3, 37.8, 28.1. HRMS (ESI): m/z 488.1675 [M-Boc+H]$^+$ (calcd for $C_{24}H_{22}N_7O_5^+$, 488.1677).

*Synthesis of probe **2**.* Compound **10** (80 mg, 0.14 mmol) was dissolved in a mixed solution (4.0 mL, CH_2Cl_2/TFA = 1/1), which was stirred for 30 min at room temperature to afford compound **11**. After removed the solvent, **11** was redissolved in anhydrous CH_2Cl_2 (10 mL), compound **7** (63 mg, 0.25 mmol), EDCI (96 mg, 0.5 mmol) and DMAP (122 mg, 1.0 mmol) were added. After stirred for 3 h, solvent was removed under reduced pressure, and the residue was subjected to silica gel column chromatography with CH_2Cl_2/CH_3OH (1000/8) to get a red solid **2** (86 mg, 88%). ^{1}H NMR (400 MHz, $CDCl_3$) δ 8.59 (d, J = 7.2 Hz, 1H), 8.52 (d, J = 8.1 Hz, 1H), 8.49 (d,

J = 8.7 Hz, 1H), 8.40 (d, *J* = 8.8 Hz, 1H), 7.77 (t, *J* = 7.8 Hz, 1H), 7.31 (d, *J* = 8.0 Hz, 1H), 6.42 (d, *J* = 8.9 Hz, 1H), 6.19 (t, *J* = 5.1 Hz, 1H), 4.45 - 4.35 (m, 4H), 4.35 - 4.30 (m, 2H), 3.60 - 3.52 (m, 6H), 2.73 (s, 2H), 1.97 (s, 3H), 1.84 (s, 3H), 1.79 (s, 3H), 1.30 (s, 6H); $^{13}C\{^1H\}$ NMR (101 MHz, $CDCl_3$) δ 191.2, 187.6, 172.2, 164.9, 164.5, 154.8, 153.8, 145.2, 145.0, 144.7, 143.9, 137.5, 137.2, 135.0, 132.8, 131.8, 130.1, 130.0, 126.5, 126.2, 124.5, 123.2, 117.6, 115.6, 103.4, 52.6, 49.5, 49.2, 39.4, 38.1, 28.7, 14.1, 12.7, 12.1. HRMS (ESI): m/z 720.2772 $[M+H]^+$ (calcd for $C_{38}H_{38}N_7O_8^+$, 720.2776).

Spectroscopic studies

Spectroscopic experiments were normally performed in phosphate buffered saline (PBS, 50 mM, pH 7.4 containing 0.007% BSA, 100 µM NADH and 10% DMSO) at room temperature. Probes **1** and **2** were dissolved into DMSO to prepare the stock solution with a concentration of 5.0 mM. Probes were diluted in PBS buffer to afford the final concentration of 1-10 µM. All measurements were performed in a 3 mL cuvette with 2 mL of solution.

For the absorbance studies, probes (10 µM) were incubated with H_2S (1 mM) and hNQO1 (5 µg/mL) simultaneously or separately. The absorbance profiles were recorded at different time points.

For the time-dependent fluorescence response of probes **1** and **2** toward H_2S and/or hNQO1, the probe (1 µM) was incubated with H_2S (200 µM) and hNQO1 (1 µg/mL) simultaneously or separately. The fluorescence profiles were recorded at different time points within 2 h. For the assessment on sensitivity of **2**, the probe (1 µM) was first treated with different concentrations of H_2S (0-200 µM) in the presence of hNQO1 (1 µg/mL) for 2 h. Subsequently, **2** (1 µM) was incubated with various levels of hNQO1 (0-1 µg/mL) in the presence of H_2S (50 µM) for 2 h. For the investigation on selectivity of **2**, the probe (1 µM) was separately incubated with some RSS (SO_3^{2-}, 200 µM; $S_2O_3^{2-}$, 200 µM), biothiols (Cys, 500 µM; Hcy, 500 µM; GSH, 5 mM) and ROS (H_2O_2, 200 µM; HClO, 200 µM) in the presence of hNQO1 (1 µg/mL) or H_2S (200 µM) for 2 h before recording the emission profiles.

Cell culture

HT29 cells (human colorectal epithelial cancer cell line) were grown in DME/F-12 supplemented with FBS (10%), penicillin (100 U mL^{-1}) and streptomycin (100 U mL^{-1}). HCT116 cells (human colorectal epithelial cancer cell line) were grown in McCoy's 5A with FBS (10%), penicillin (100 U mL^{-1}) and streptomycin (100 U mL^{-1}). FHC cells (human normal colorectal epithelial cells line) were grown in RPMI 1640 supplemented with FBS (10%), penicillin (100 U mL^{-1}) and streptomycin (100 U mL^{-1}). HepG2 cells (human hepatocellular carcinoma cell line), HeLa cells (human cervical carcinoma cell line) and HEK293A cells (human embryonic kidney cells) were grown in high glucose DMEM (GIBICO) supplemented with FBS (10%), penicillin (100 U mL^{-1}), streptomycin (100 U mL^{-1}) and L-glutamine (4 mM). All cells were maintained in an incubator at 37 °C with 5% CO_2/air environment.

Cytotoxicity assay

The *in vitro* cytotoxicity was first measured using standard methyl thiazolyl tetrazolium (MTT, Sigma-Aldrich) assay in HT29 cell lines. Briefly, cells growing in log phase were seeded into 96 well cell-culture plate at 1×10^4/well. The probe **2** (100 µL/well) at concentrations of 0-33 µM was added to the wells of the treatment group, and 100 µL/well DMSO diluted in DMEM at final concentration of 0.5% to the negative control group, respectively. The cells were incubated for 2 h at 37 °C under 5% CO_2. The combined MTT/PBS solution was added to each well of the 96-well assay plate and incubated for an additional 4 h. An enzyme-linked immunosorbent assay (ELISA) reader (Bio-Rad Laboratories, Hercules, CA, USA) was used to measure the OD_{490} (absorbance value) of each well referenced at 490 nm. The following formula was used to calculate the viability of cell growth: viability (%T) = $A_1/A_2 \times 100\%$, where A_1 denotes absorbance value of treatment group, and A_2 denotes absorbance value of control.

The cytotoxicity of **2** was further determined by the xCELLigence RTCA S16 (ACEA Biosciences),[2] which is an instrument that uses microelectronic plates integrated with gold microelectrode arrays on glass substrate in the bottom of the

wells to measure the proliferation of adherent cells. When cells replicate and grow, they can cover the electrosensitive elements and cause higher impedance, and the electronic current will be changed. The software connected to a computer can real-time monitor these changes, reflecting the proliferation of adherent cells. Briefly, HEK293A cells growing in log phase were seeded into 16-well cell-culture plate (100 μL/well). When the amount of cells reached 5 × 10^4/well, probe **2** was added into each well with final concentration as: 0, 1, 2, 5, 10, 15 and 20 μM. The cells were incubated at 37 °C under 5% CO_2, and data were collected every 15 min for the druation of the assay.

Confocal fluorescence imaging for living cells

Cells were maintained in exponential growth phase, and then seeded in a glass-bottom 35 mm plate (~ 2×10^4 cells per well). For the bioimaging of probe **2**, cells were excited by a 405 nm laser diode and detected at BA = 500-600 nm. As for the bioimaging of control probes **NIR-H$_2$S** and **NIR-hNQO1**, cells were excited by a 635 nm laser diode and detected at BA = 655-755 nm. The cell experiments were performed in the presence of 0.2% DMSO (2 μL of 5 mM probe **2** was added into 1 mL culture medium).

For the endogenous H_2S and hNQO1 imaging in HT29, HepG2, FHC, HCT116 and HeLa cells, cells were treated with probe **2** (10 μM) at 37 °C for 1 h, washed by PBS twice, and then imaged. For inhibitor-treated bioimaging, cells were pretreated with AOAA (200 μM) or dicoumarol (100 μM) for 30 min, then incubated with probe **2** (10 μM) for 1 h. After being washed with PBS twice, the cells were imaged. For H_2O_2-induced bioimaging, HeLa cells were co-incubated with probe **2** (10 μM) and H_2O_2 (50 μM) for 1 h, washed with PBS twice before imaging. In the control experiments, cells were pretreated with AOAA (200 μM) or dicoumarol (100 μM) for 30 min, then co-incubated with probe **2** (10 μM) and H_2O_2 (50 μM) for 1 h, washed and imaged. Additionally, after co-incubation with H_2O_2 (50 μM) and **2** (10 μM) for 1 h, AOAA-pretreated cells was further treated with Na_2S (150 μM) for 30 min, then washed with PBS twice before imaging. Random locations on each plate were

selected for imaging, then fluorescent intensity of these images were determined by software Image-Pro Plus. The results were expressed as the mean ± standard deviation. Comparisons between two sets of data were determined by Student's *t-test*. For these tests, $P < 0.05$ or $P < 0.01$ was regarded as statistically significant. To present more apparent contrast, we normalized the data based on the lowest fluorescence intensity in each experiment.

References

1. C. Zhang, B.-B. Zhai, T. Peng, Z. Zhong, L. Xu, Q.-Z. Zhang, L.-Y. Li, L. Yi and Z. Xi, *Dyes Pigments*, 2017, **143**, 245.

2. Y. A. Abassi, B. Xi, W. Zhang, P. Ye, S. L. Kirstein, M. R. Gaylord, S. C. Feinstein, X. Wang and X. Xu, *Chem. Biol.*, 2009, **16**, 712.

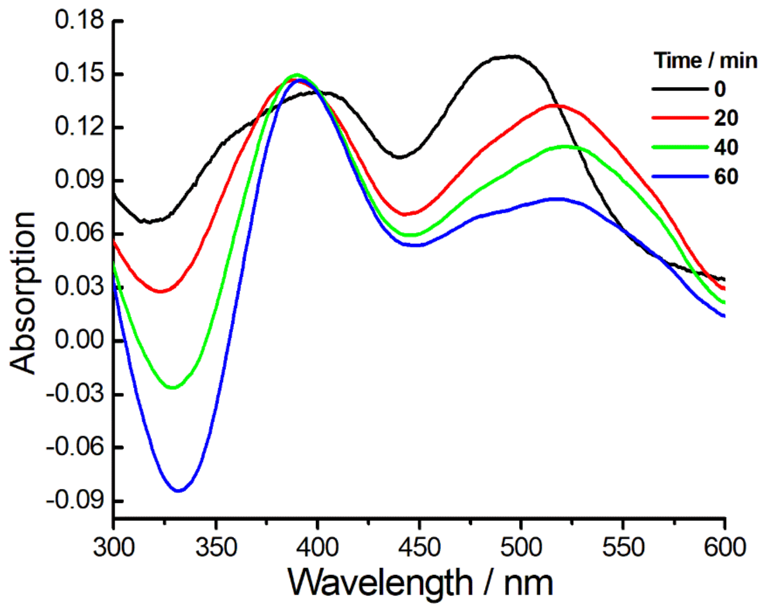

Figure S1. Time-dependent UV-vis spectra of probe **1** (10 μM) towards H_2S (1 mM) and hNQO1 (5 μg/mL) in PBS buffer (50 mM, pH 7.4 containing 0.007% BSA, 100 μM NADH and 10% DMSO).

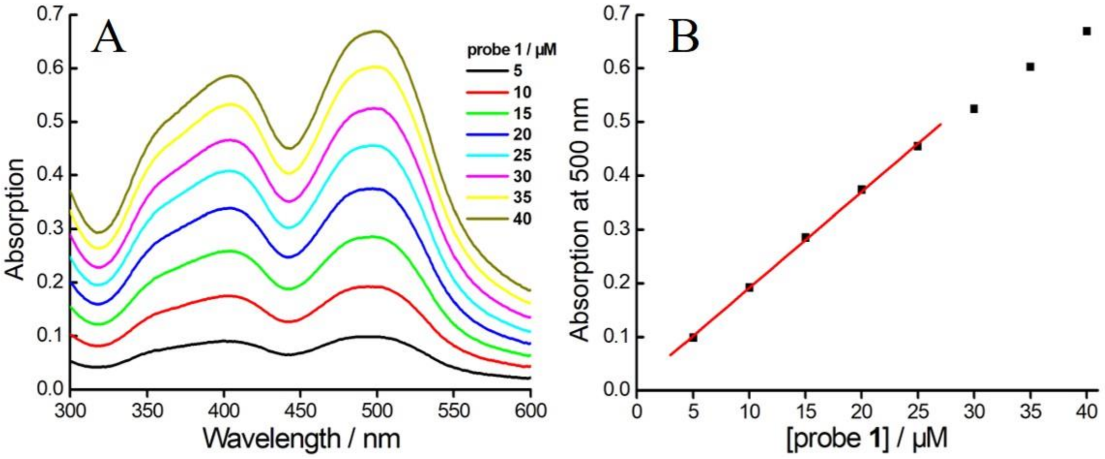

Figure S2. (A) UV-vis spectra of probe **1** at varied concentrations (0-40 µM) in PBS buffer (50 mM, pH 7.4 containing 0.007% BSA, 100 µM NADH and 0.2% DMSO). (B) Linear relationship between absorptions at 500 nm of **1** and the probe concentrations (R^2 = 0.998).

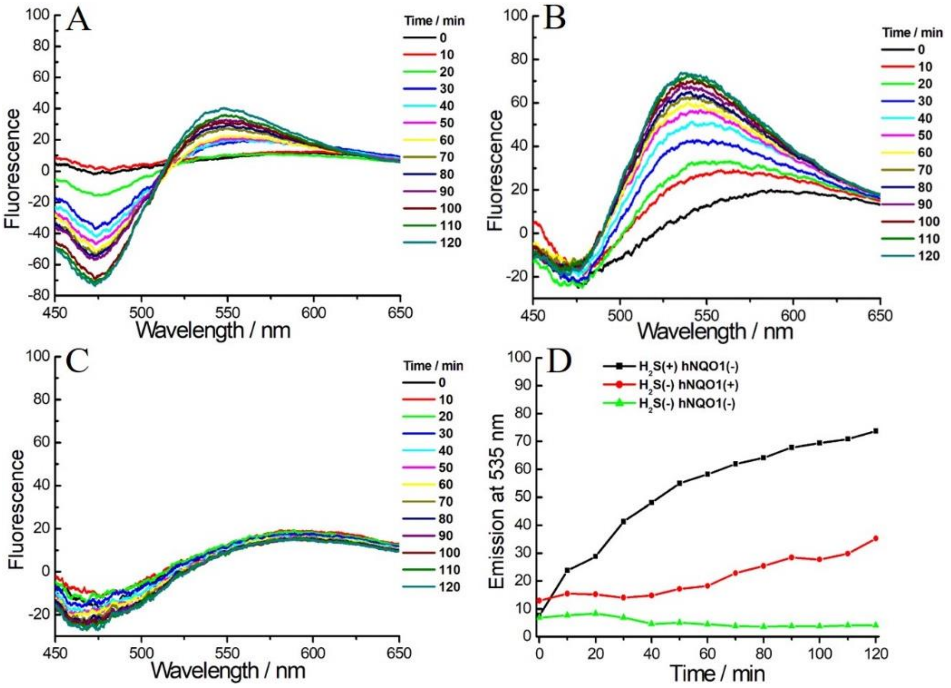

Figure S3. Time-dependent fluorescent response of probe **2** (1 µM) toward H_2S (200 µM) or hNQO1 (1 µg/mL). **2** reacted with hNQO1 (A) or H_2S (B). (C) Stability test of **2** in PBS buffer within 2 h. (D) Time-dependent emissions of **2** at 535 nm in the presence or absence of H_2S/hNQO1.

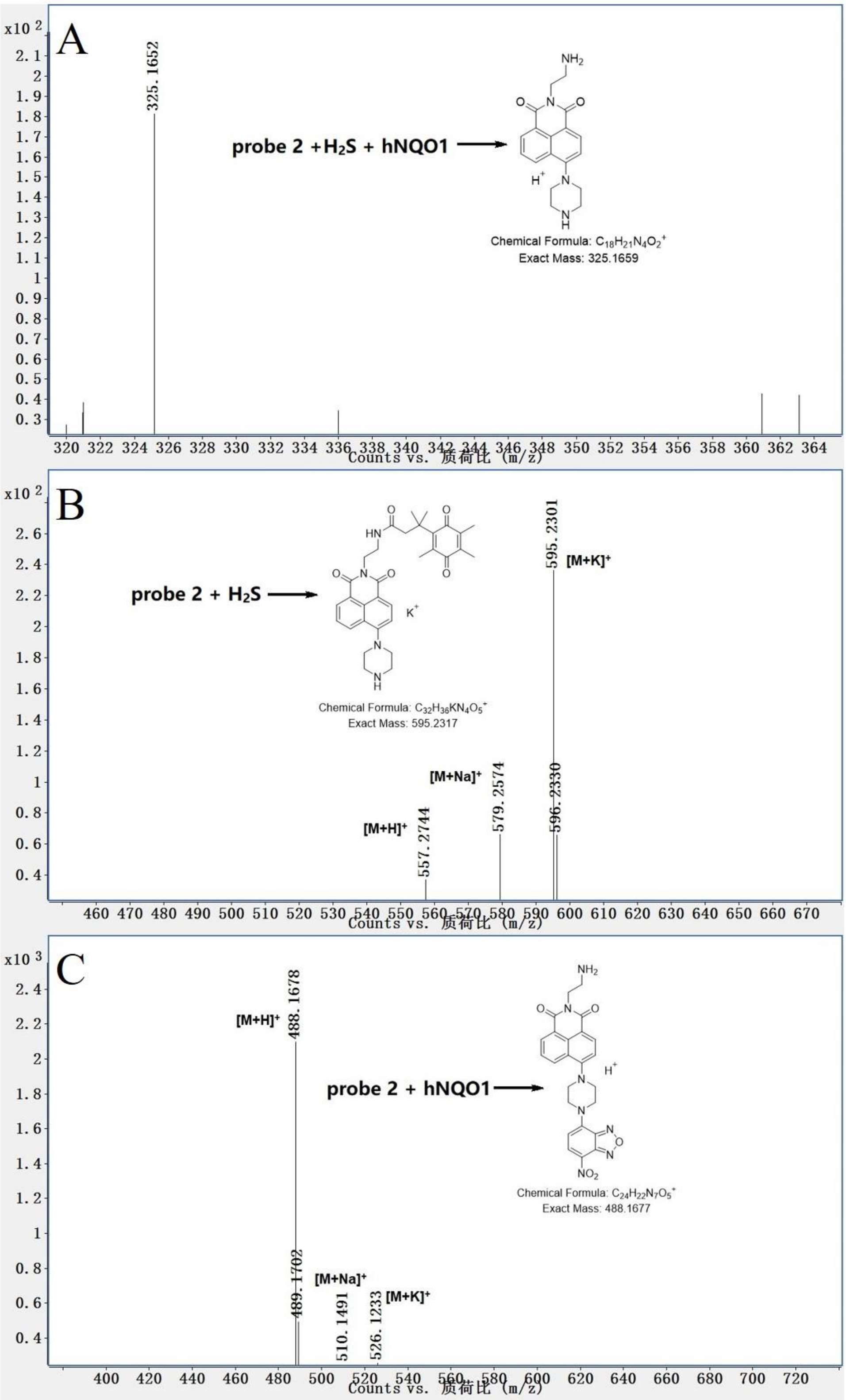

Figure S4. HRMS spectra of the products of probe **2** incubated with H_2S and hNQO1 simultaneously (A) or separately (B, H_2S; C, hNQO1). Probe **2** (100 μM) was treated by H_2S (1.5 mM) and hNQO1 (20 μg/mL) simultaneously or separately in PBS buffer (50 mM, pH 7.4 containing 0.007% BSA, 500 μM NADH and 30% CH_3CN) overnight at room temperature.

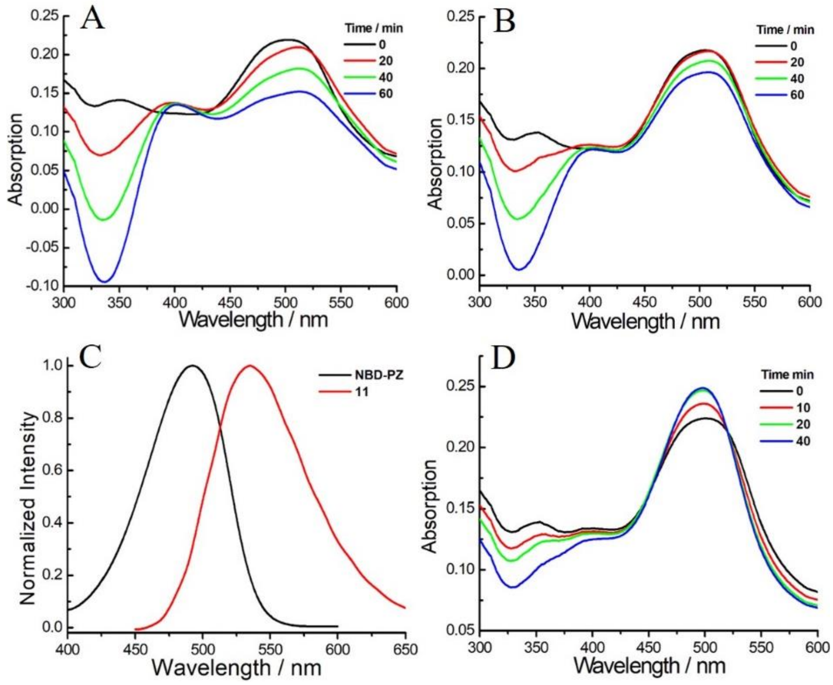

Figure S5. Time-dependent UV-vis spectra of probe **2** (10 µM) towards H_2S (1 mM)/hNQO1 (5 µg/mL) in PBS buffer (50 mM, pH 7.4 containing 0.007% BSA, 100 µM NADH and 10% DMSO). (A) **2** was treated with H_2S and hNQO1 simultaneously. **2** reacted with H_2S (B) or hNQO1 (D) alone. (C) Normalized absorption spectrum of NBD-PZ and emission spectrum of **11** in PBS buffer containing 10% DMSO.

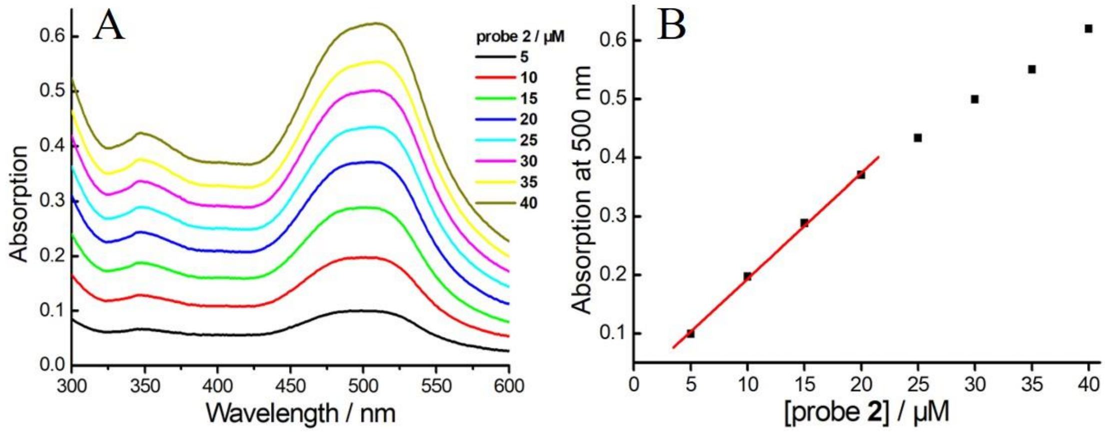

Figure S6. (A) UV-vis spectra of probe **2** at varied concentrations (0-40 µM) in PBS buffer (50 mM, pH 7.4 containing 0.007% BSA, 100 µM NADH and 0.2% DMSO). (B) Linear relationship between absorptions at 500 nm of **2** and probe concentrations (R^2 = 0.999).

Compound	Absorption peak / nm	Emission peak/ nm	Quantum yield
**1**	405/500	/	0.15%
**2**	350/500	/	0.041%
**11**	400	535	7.0%

Table S1. Optical properties of compounds **1**, **2** and **11** in PBS buffer (50 mM, pH 7.4 containing 0.007% BSA, 100 µM NADH and 10% DMSO).

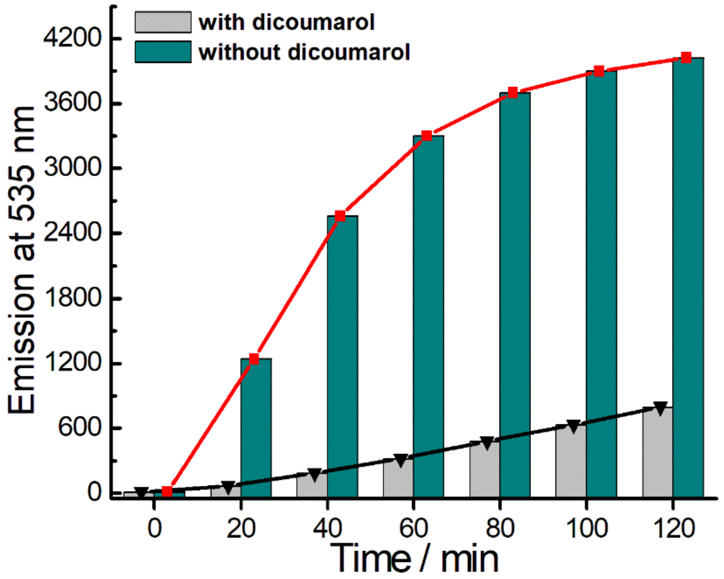

Figure S7. Time-dependent fluorescence emissions of **2** at 535 nm toward H_2S (200 μM) and hNQO1 (5 μg/mL) in PBS buffer (50 mM, pH 7.4 containing 0.007% BSA, 100 μM NADH and 10% DMSO) in the presence or absence of inhibitor dicoumarol (100 μM).

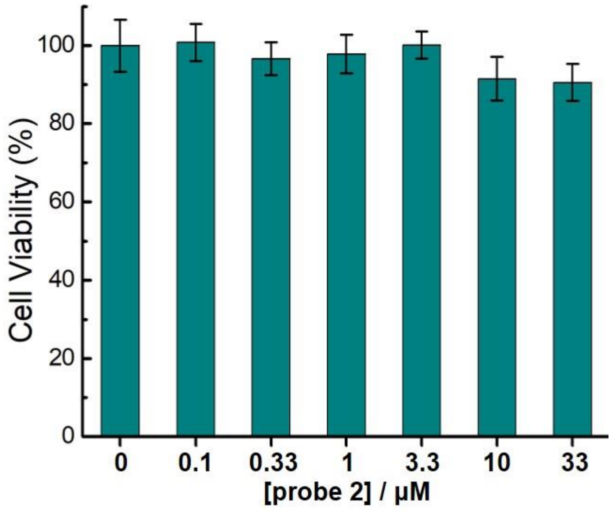

Figure S8. Cell viability (%) estimated by MTT assay versus incubation concentrations of **2**.

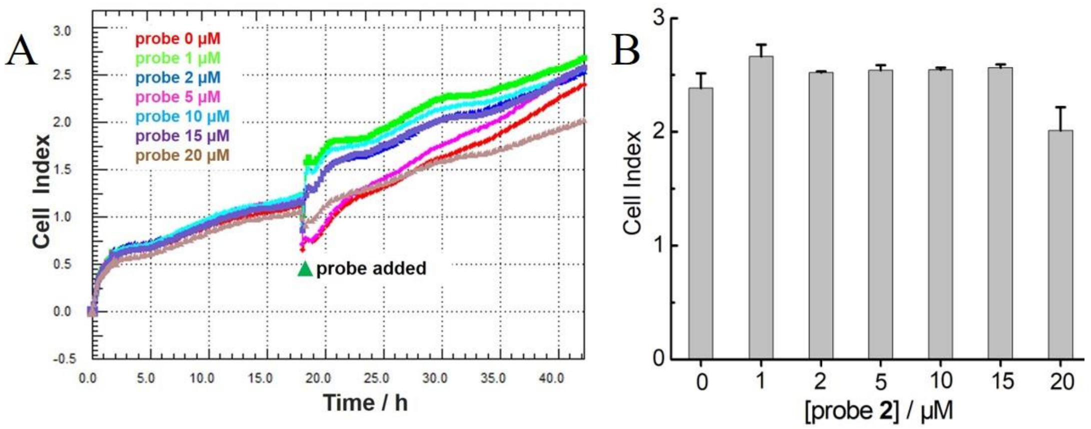

Figure S9. Evaluation on the cytotoxicity of **2** using xCELLigence RTCA system. (A) Real-time monitoring of the density-dependent growth and proliferation of HEK293A cells with different probe concentration. (B) The cell index values versus probe concentration at the time point of 24 h.

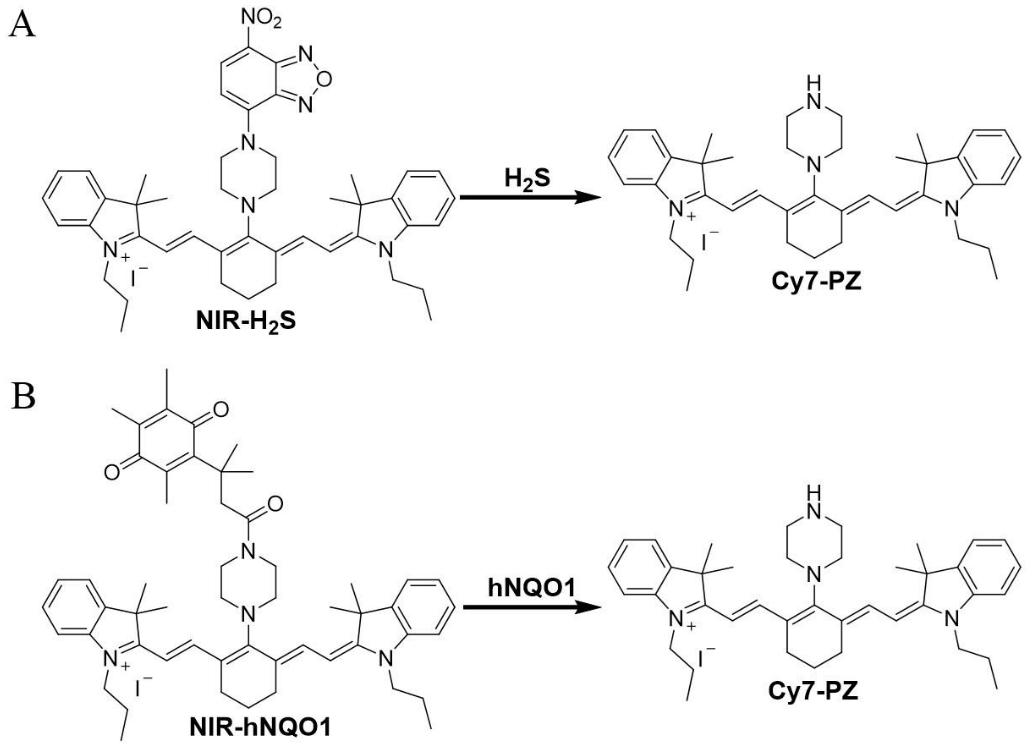

Figure S10. (A) Chemical structure of control probe **NIR-H$_2$S** and its reaction with H_2S. (B) Chemical structure of control probe **NIR-hNQO1** and its reaction with hNQO1.

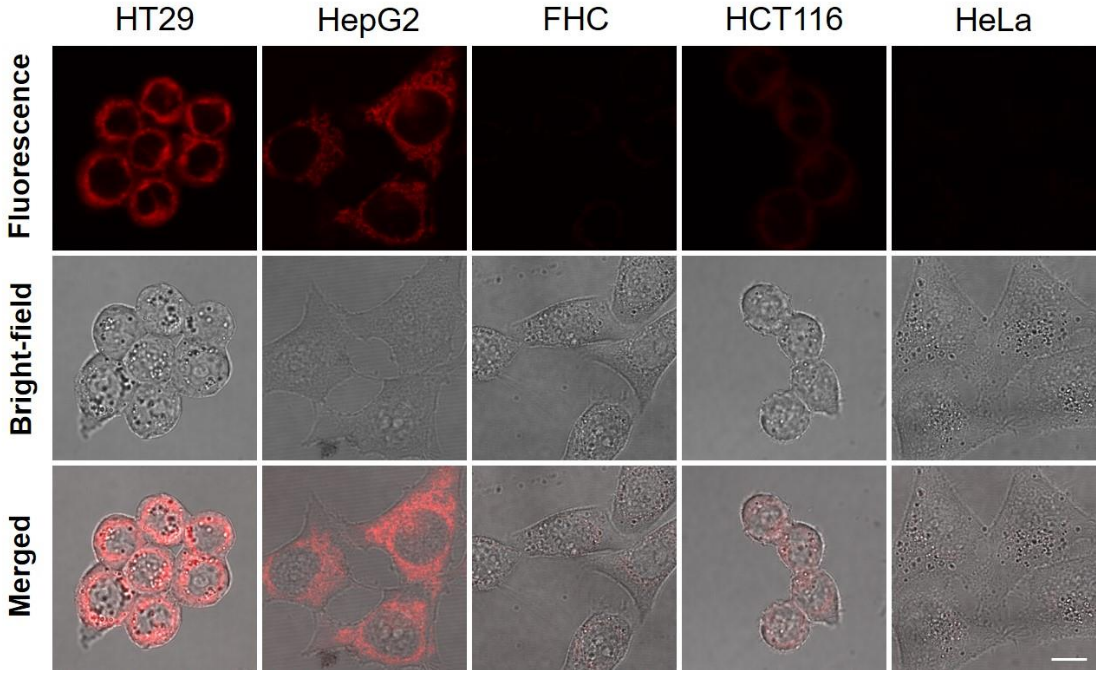

Figure S11. Confocal fluorescence bioimaging of single-reaction probe **NIR-H_2S**. Cells were incubated with **NIR-H_2S** (10 μM) for 30 min, washed and imaged. Scale bar, 10 μm.

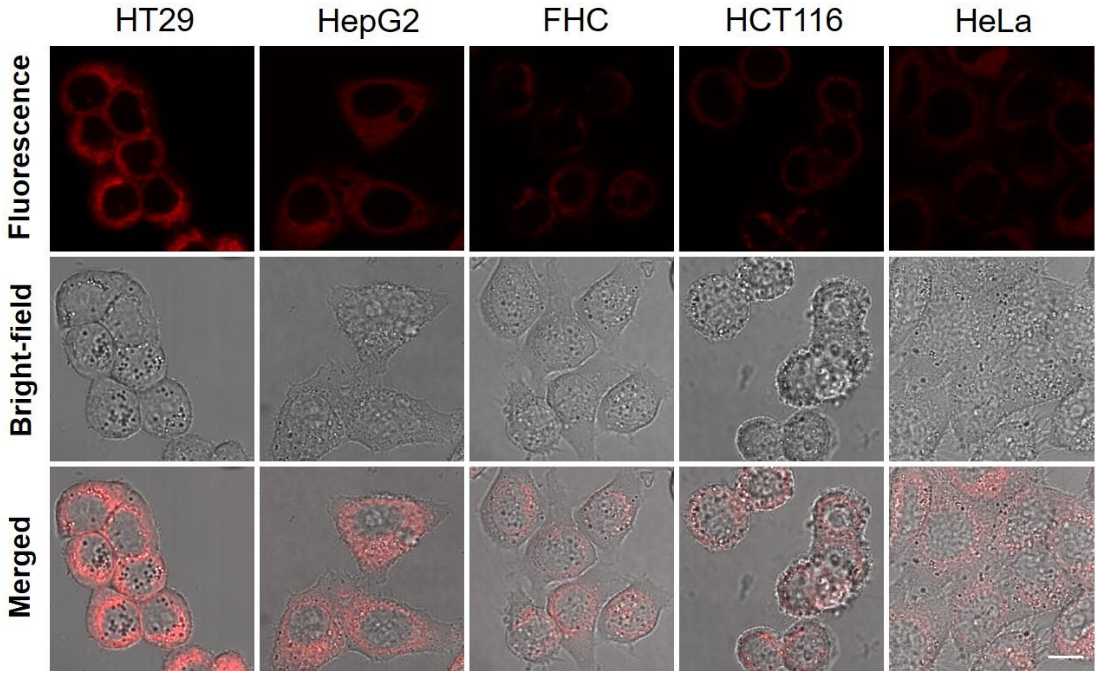

Figure S12. Confocal fluorescence bioimaging of single-reaction probe **NIR-hNQO1**. Cells were **NIR-hNQO1** (10 μM) for 20 min, washed and imaged. Scale bar, 10 μm.

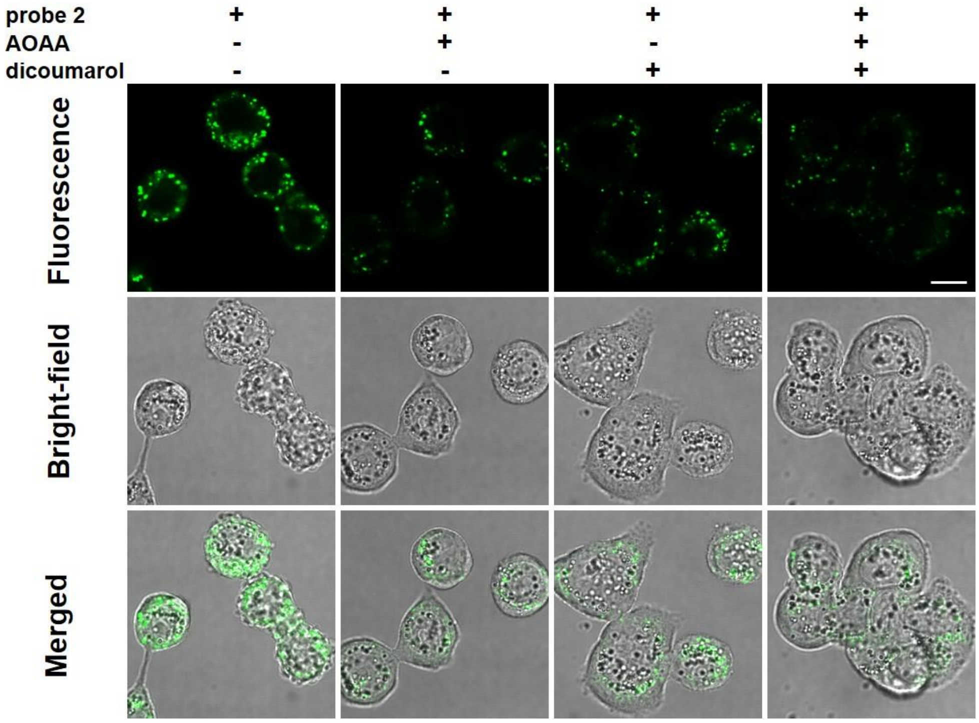

Figure S13. Confocal microscopy images for HT29 cells using **2** in the presence of enzymatic inhibitors. Cells were incubated with **2** (10 μM) alone for 1 h; cells were pretreated with AOAA (200 μM) and/or dicoumarol (100 μM) for 30 min, then incubated with **2** (10 μM) for 1 h. Scale bar, 10 μm.

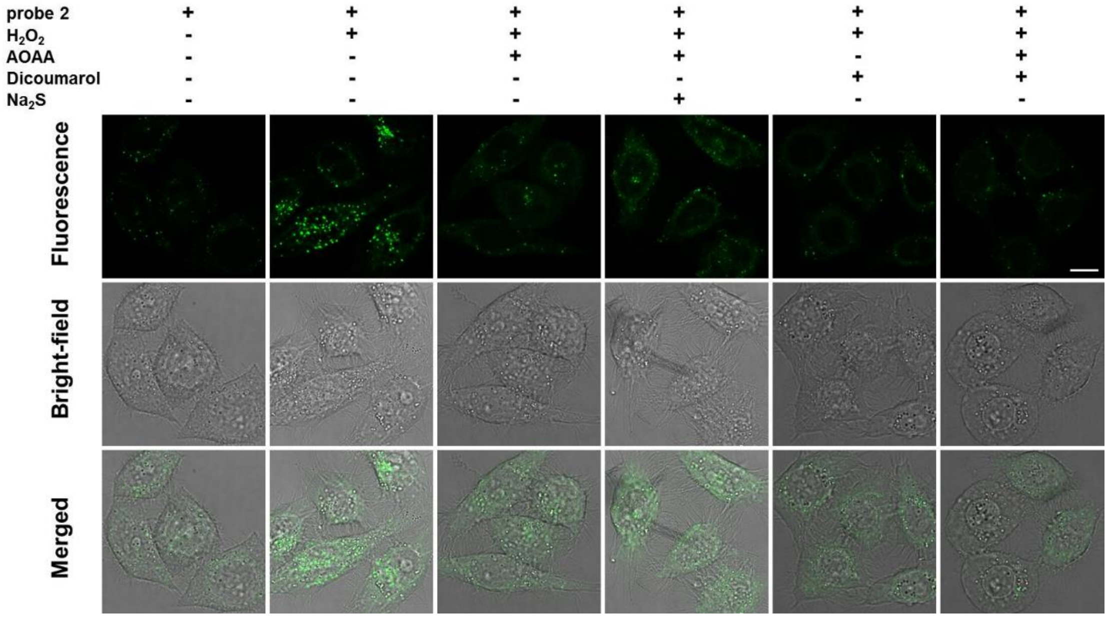

Figure S14. Confocal microscopy images for H_2O_2-induced living HeLa cells using **2**. Cells were incubated with **2** (10 μM) alone for 1 h; cells were co-incubated with **2** (10 μM) and H_2O_2 (50 μM) for 1 h. Cells were pretreated with AOAA (200 μM) and/or dicoumarol (100 μM) for 30 min, then co-incubated with **2** (10 μM) and H_2O_2 (50 μM) for 1 h. Cells were pretreated with AOAA (200 μM) for 30 min, then co-incubated with **2** (10 μM) and H_2O_2 (50 μM) for 1 h, then further with Na_2S (150 μM) for 30 min. Scale bar, 10 μm.

NMR Spectra

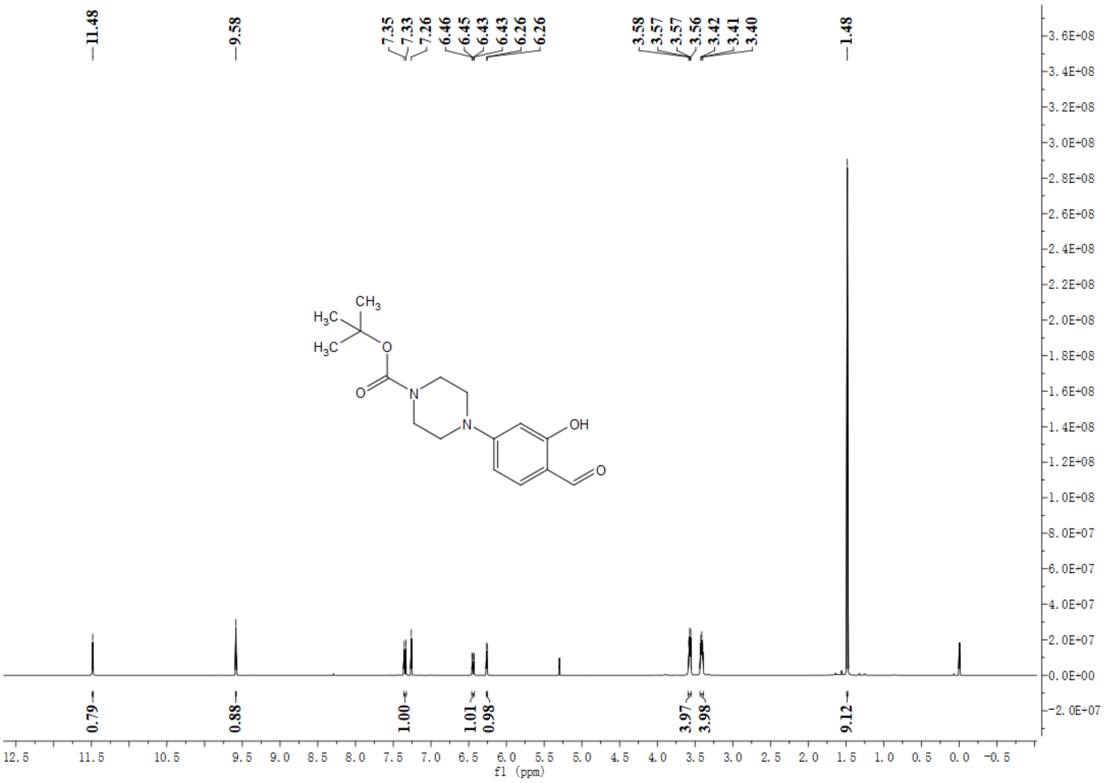

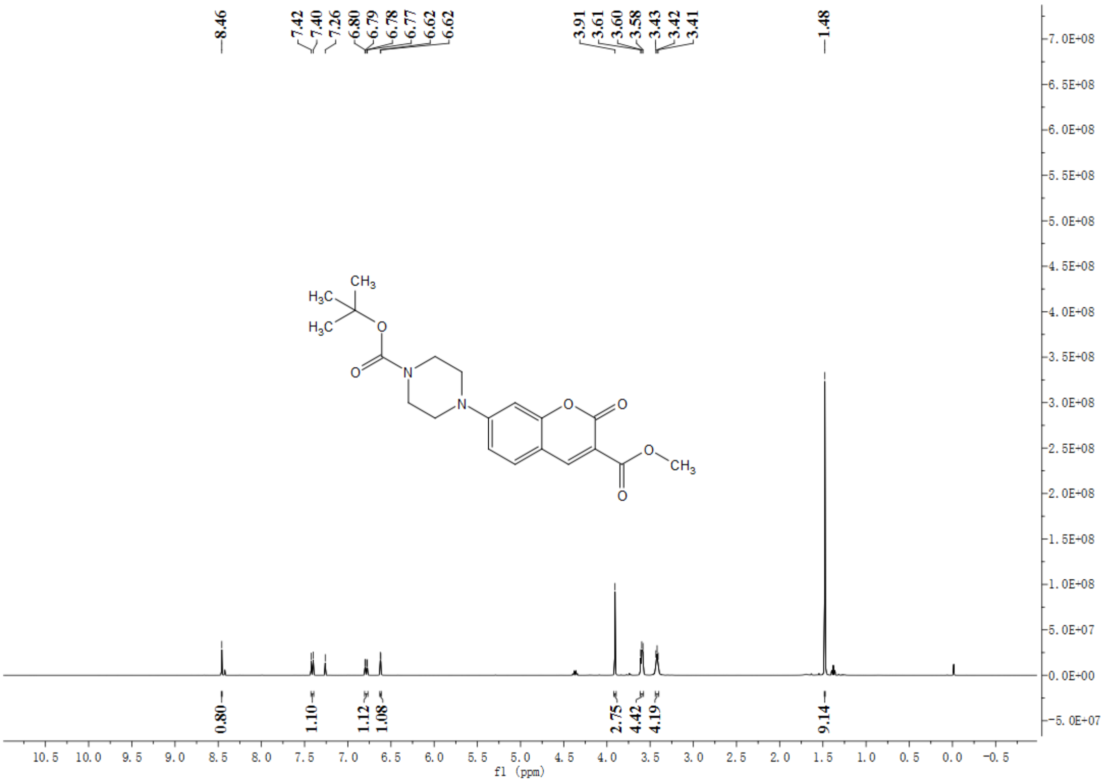

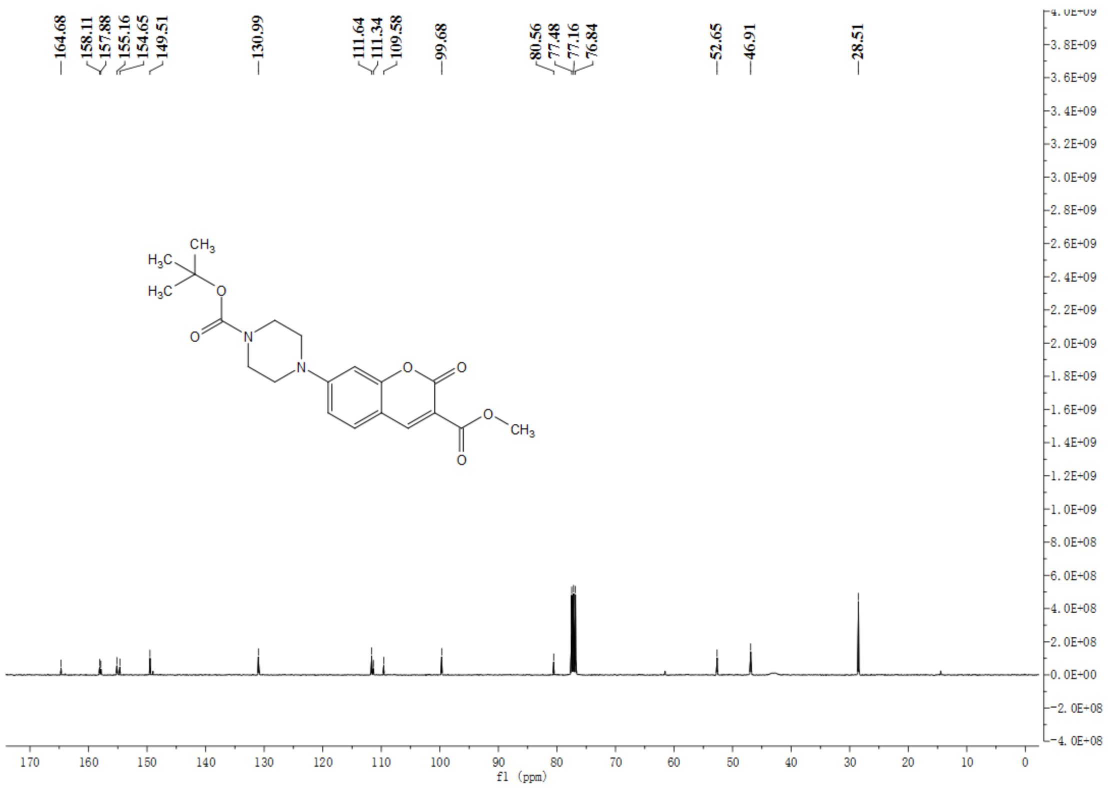

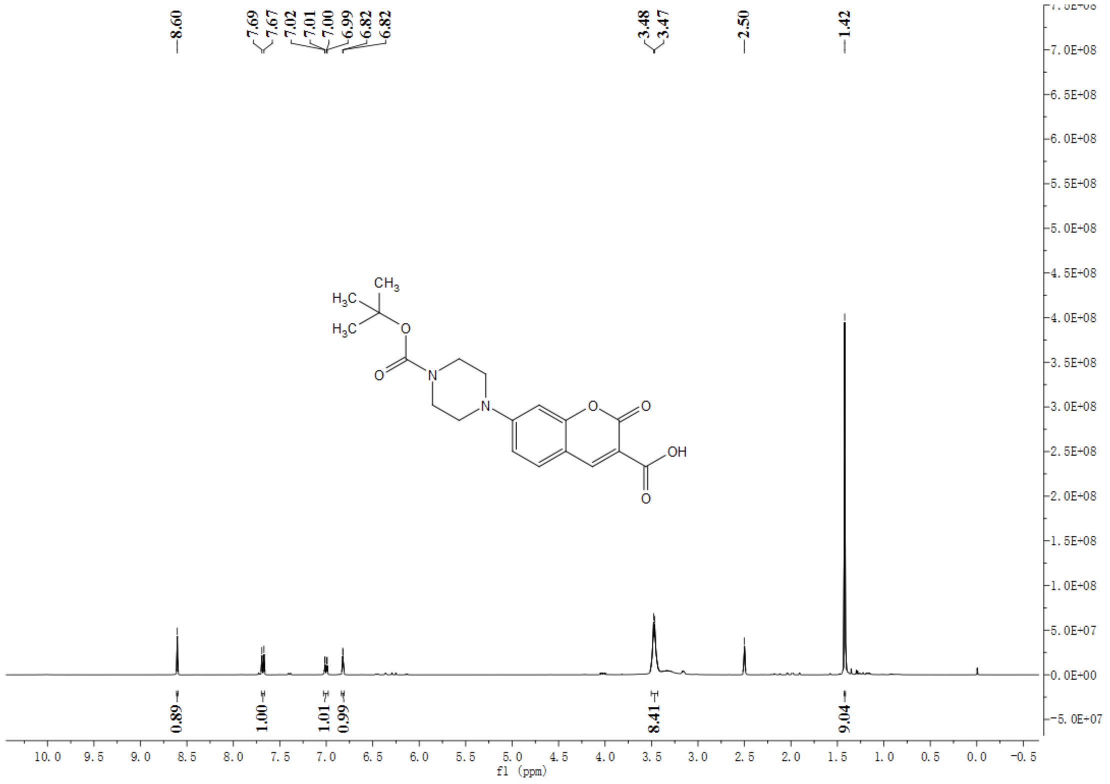

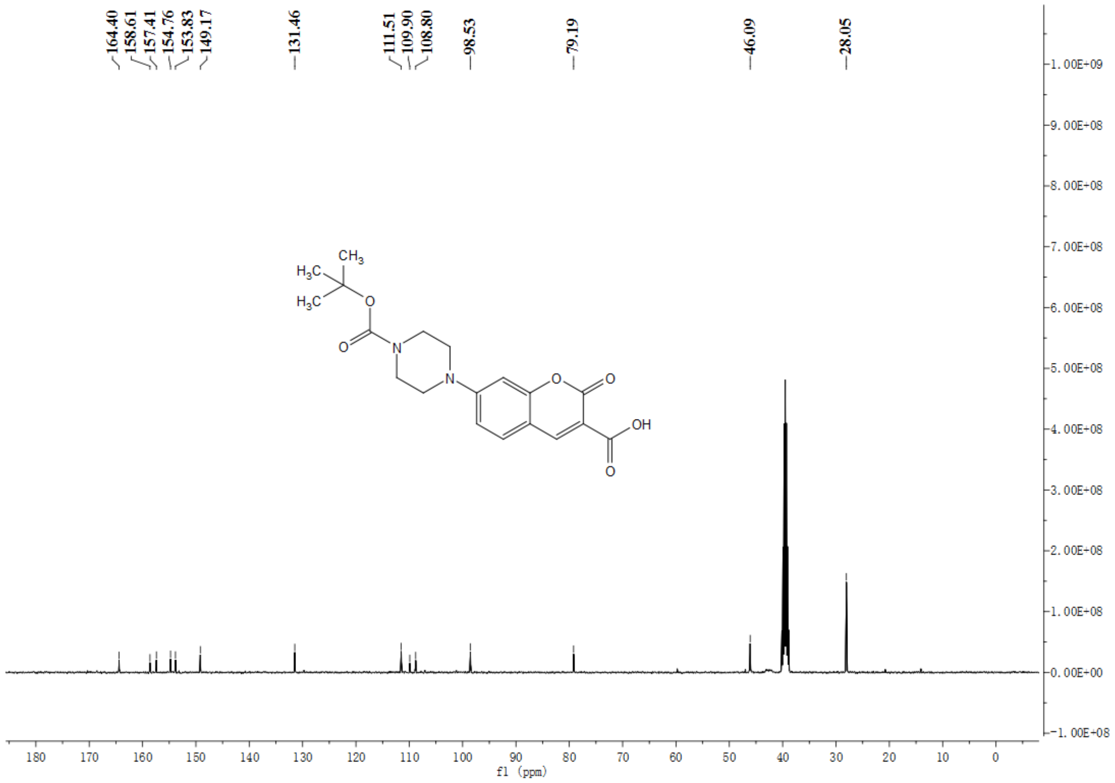
164.40
158.61
157.41
154.76
153.83
149.17
131.46
111.51
109.90
108.80
98.53
79.19
46.09
28.05
f1 (ppm)

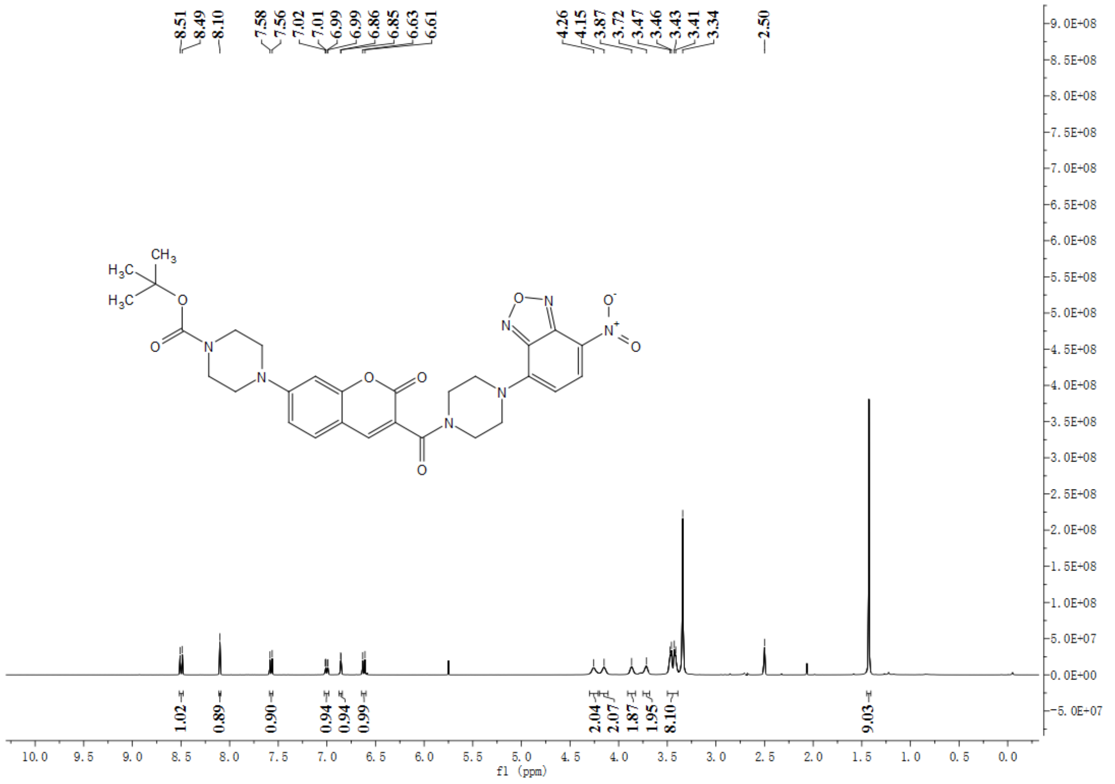
8.51
8.49
8.10
7.58
7.56
7.02
7.01
6.99
6.99
6.86
6.85
6.63
6.61
4.26
4.15
3.87
3.72
3.47
3.46
3.43
3.41
3.34
2.50
1.02
0.89
0.90
0.94
0.94
0.99
2.04
2.07
1.87
1.95
8.10
9.03
f1 (ppm)

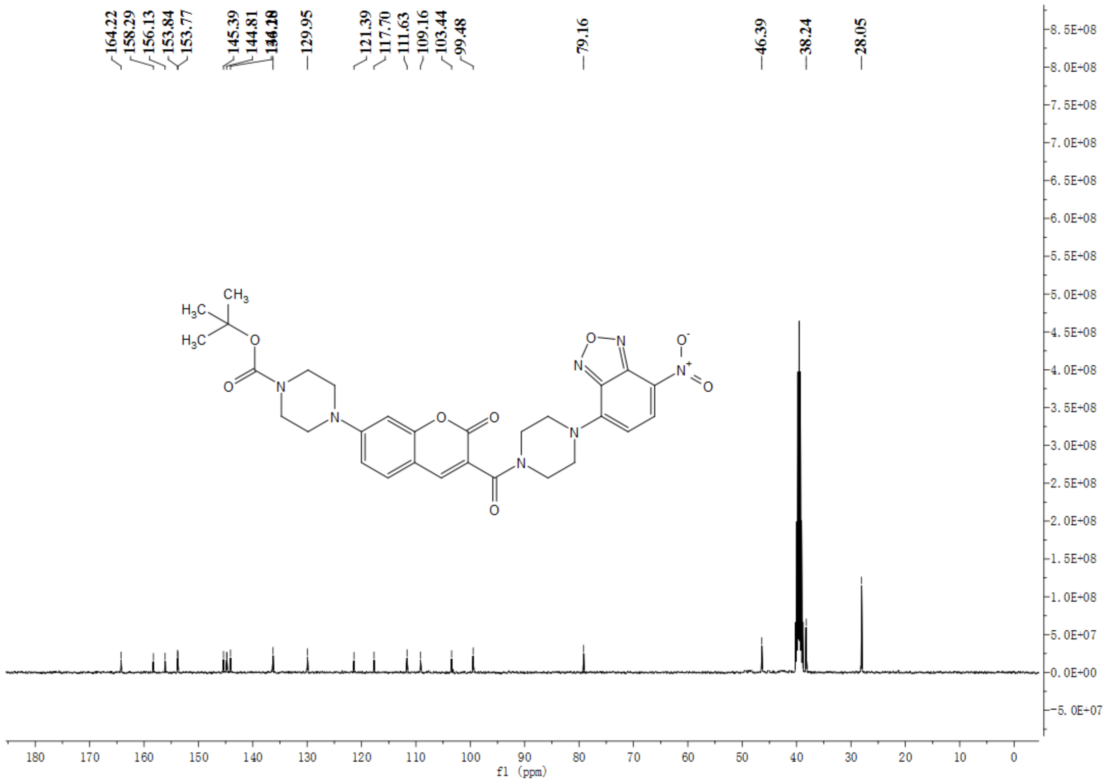
164.22
158.29
156.13
153.84
153.77
145.39
144.81
144.28
144.19
129.95
121.39
117.70
111.63
109.16
103.44
99.48
79.16
46.39
38.24
28.05
f1 (ppm)

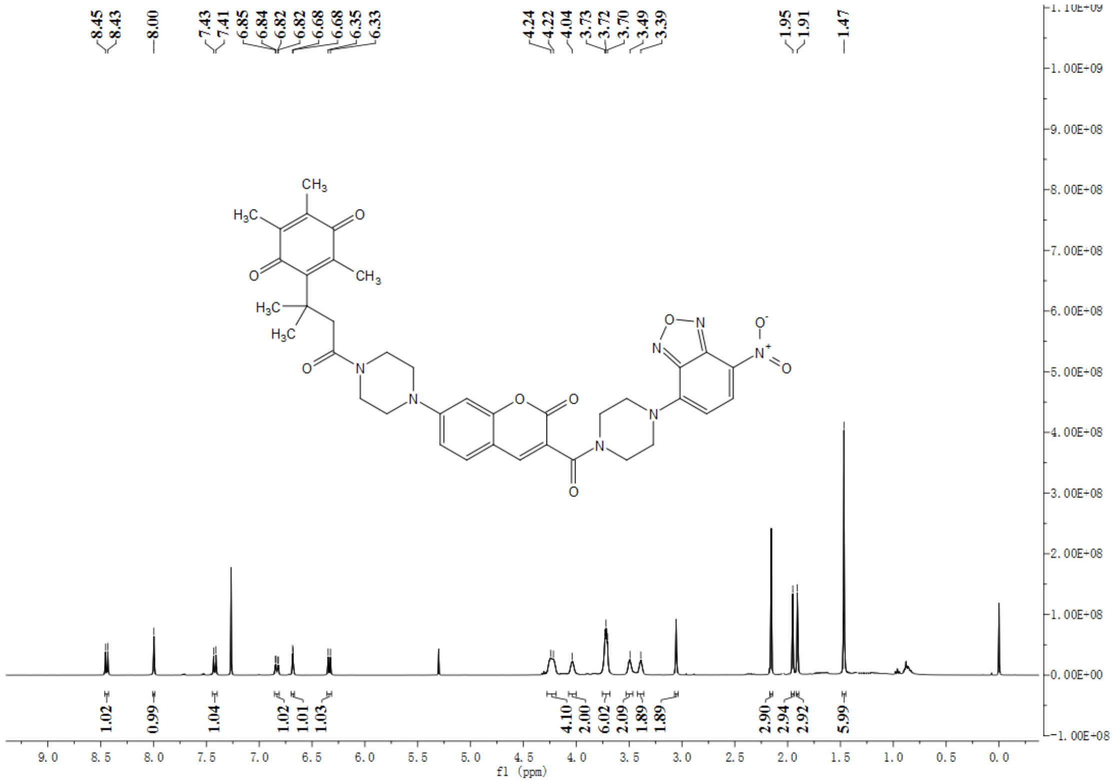
8.45
8.43
8.00
7.43
7.41
6.85
6.84
6.82
6.82
6.68
6.68
6.35
6.33
4.24
4.22
4.04
3.73
3.72
3.70
3.49
3.39
1.95
1.91
1.47
1.02
0.99
1.04
1.02
1.01
1.03
4.10
2.00
6.02
2.09
1.89
1.89
2.90
2.94
2.92
5.99
f1 (ppm)

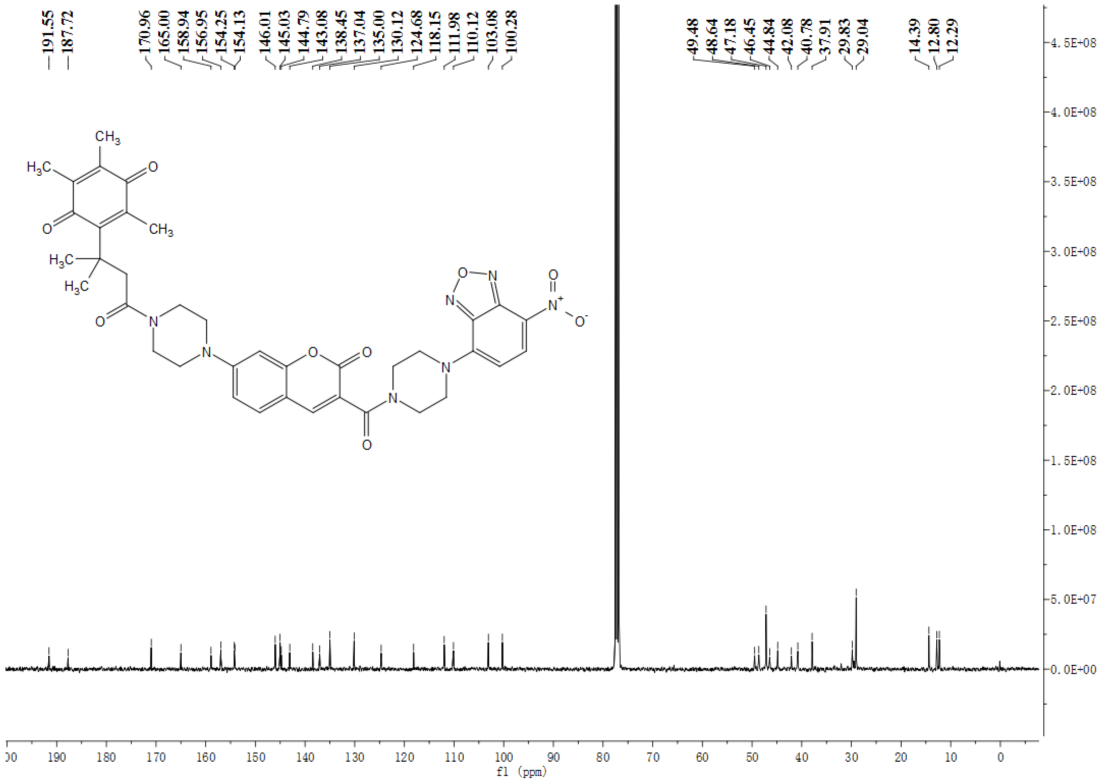
191.55
187.72
170.96
165.00
158.94
156.95
154.25
154.13
146.01
145.03
144.79
143.08
138.45
137.04
135.00
130.12
124.68
118.15
111.98
110.12
103.08
100.28
49.48
48.64
47.18
46.45
44.84
42.08
40.78
37.91
29.83
29.04
14.39
12.80
12.29
f1 (ppm)

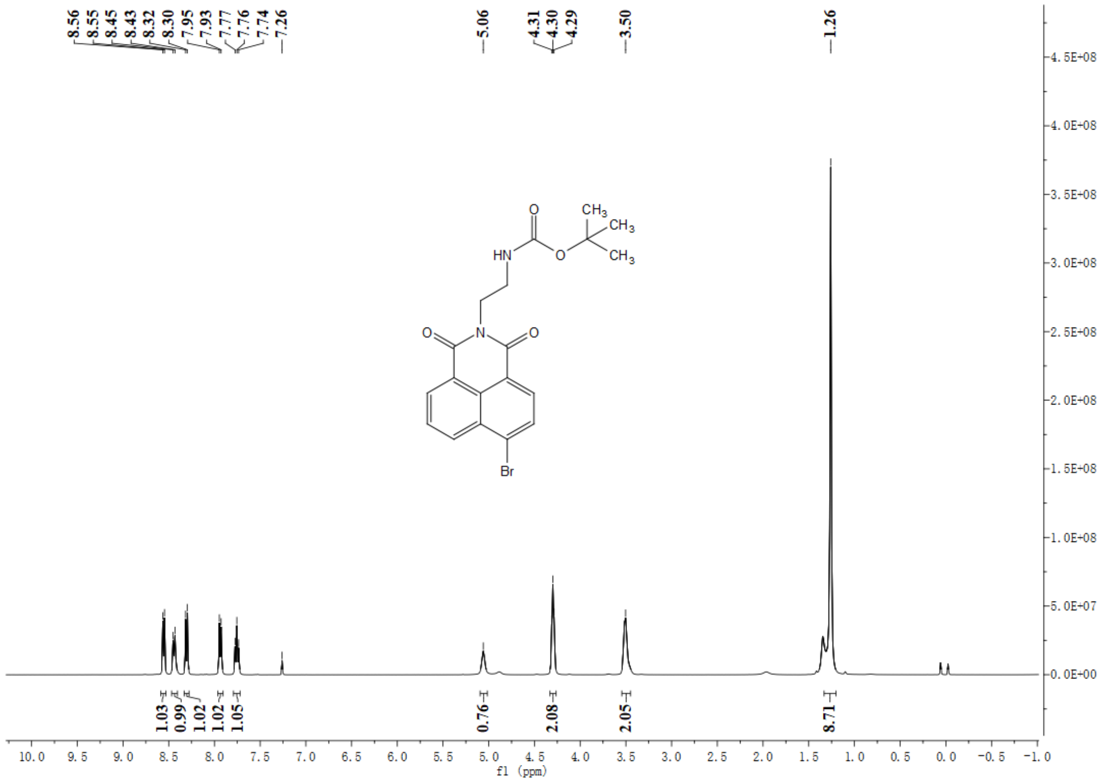
8.56
8.55
8.45
8.43
8.32
8.30
7.95
7.93
7.77
7.76
7.74
7.26
5.06
4.31
4.30
4.29
3.50
1.26
1.03
0.99
1.02
1.02
1.05
0.76
2.08
2.05
8.71
f1 (ppm)

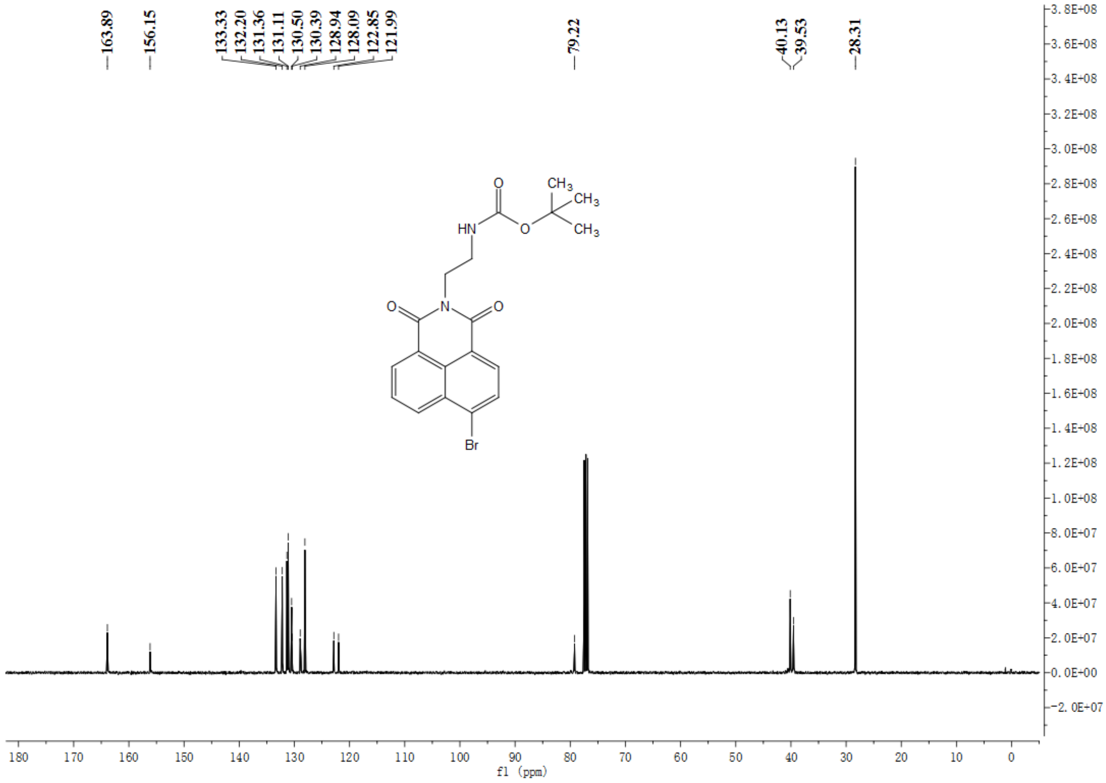
163.89
156.15
133.33
132.20
131.36
131.11
130.50
130.39
128.94
128.09
122.85
121.99
79.22
40.13
39.53
28.31
HN
O
CH3
CH3
CH3
N
Br
f1 (ppm)

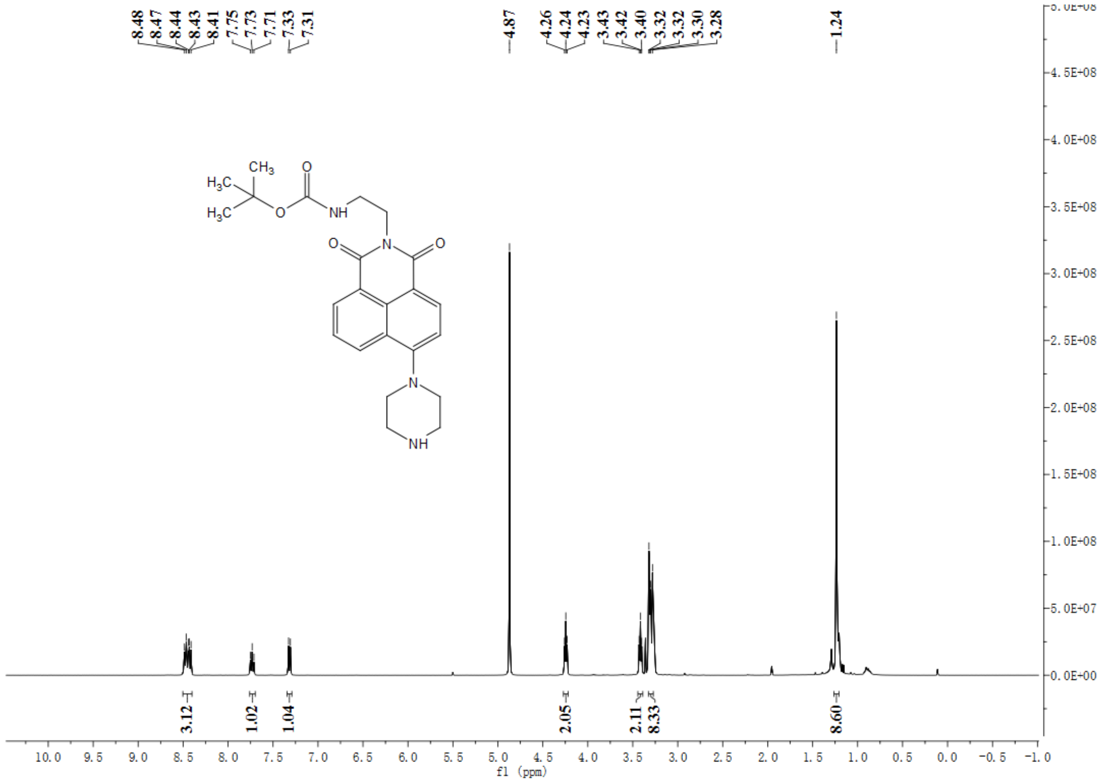
8.48
8.47
8.44
8.43
8.41
7.75
7.73
7.71
7.33
7.31
4.87
4.26
4.24
4.23
3.43
3.42
3.40
3.32
3.32
3.30
3.28
1.24
H3C
H3C
CH3
O
NH
N
3.12
1.02
1.04
2.05
2.11
8.33
8.60
f1 (ppm)

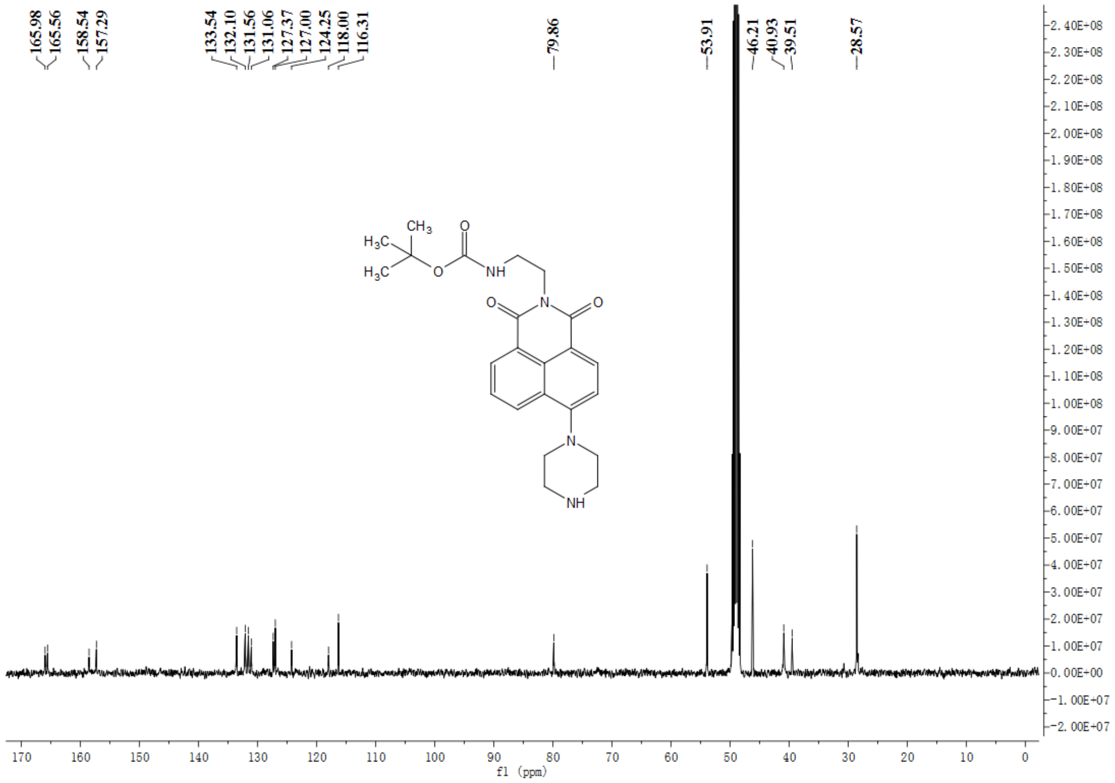
165.98
165.56
158.54
157.29
133.54
132.10
131.56
131.06
127.37
127.00
124.25
118.00
116.31
79.86
53.91
46.21
40.93
39.51
28.57
CH3
H3C
H3C
O
NH
O
N
O
NH
f1 (ppm)

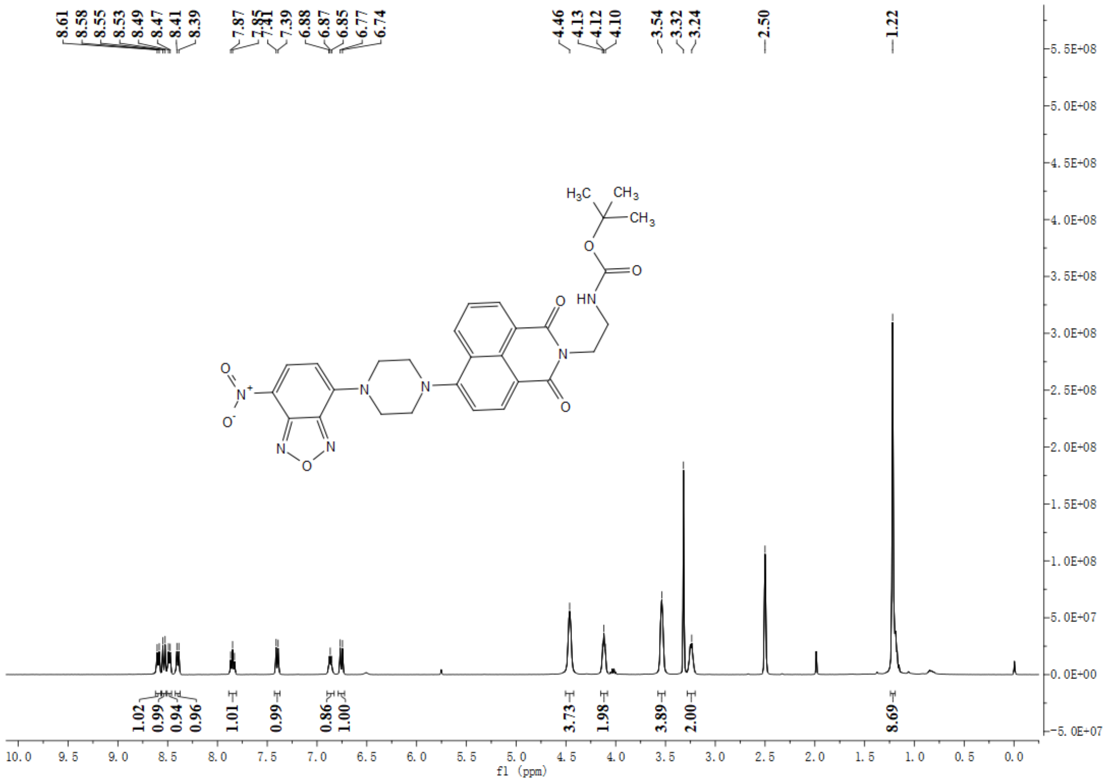
8.61
8.58
8.55
8.53
8.49
8.47
8.41
8.39
7.87
7.85
7.41
7.39
6.88
6.87
6.85
6.77
6.74
4.46
4.13
4.12
4.10
3.54
3.32
3.24
2.50
1.22
1.02
0.99
0.94
0.96
1.01
0.99
0.86
1.00
3.73
1.98
3.89
2.00
8.69
H3C
CH3
CH3
O
O
HN
O
N
O
N
N
N+
O-
N
N
O
f1 (ppm)

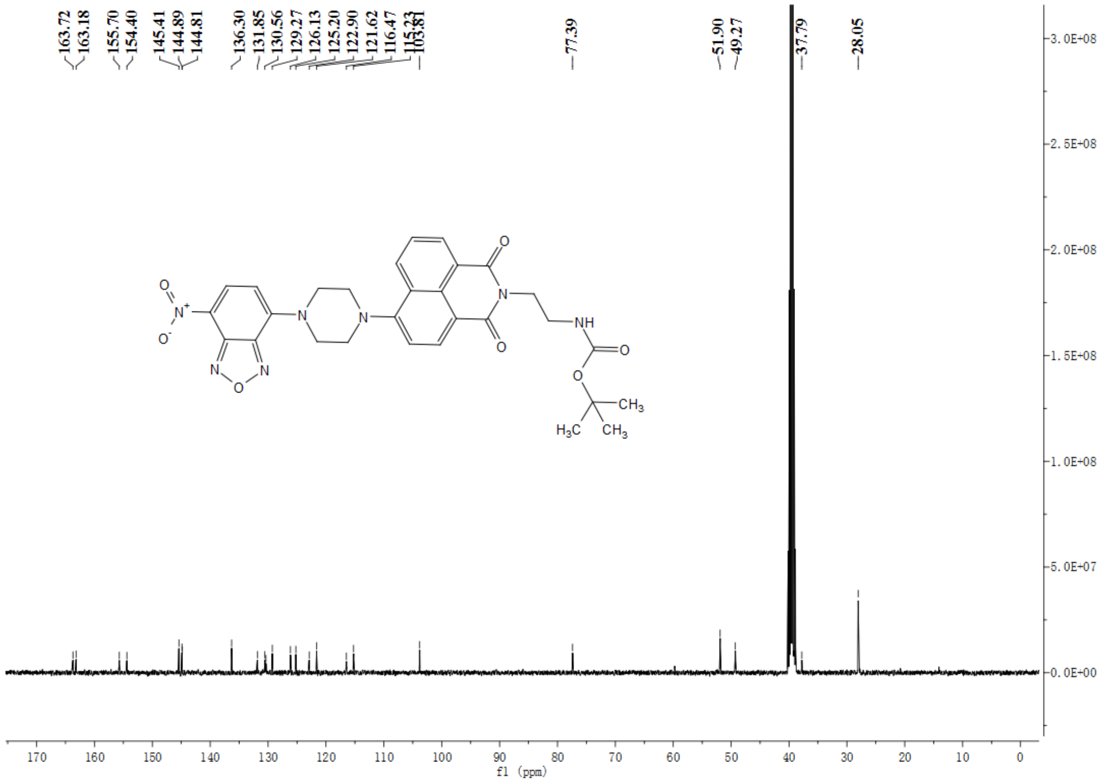
163.72
163.18
155.70
154.40
145.41
144.89
144.81
136.30
131.85
130.56
129.27
126.13
125.20
122.90
121.62
116.47
115.23
103.81
77.39
51.90
49.27
37.79
28.05
f1 (ppm)

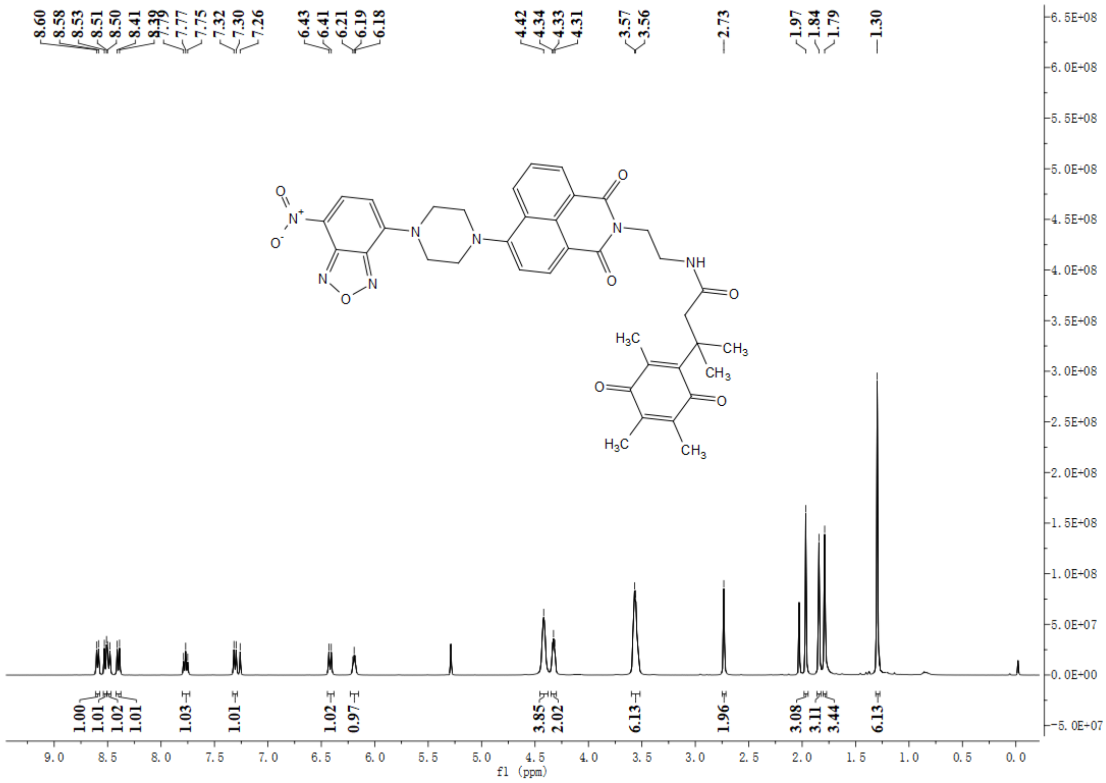
8.60
8.58
8.53
8.51
8.50
8.41
8.39
7.79
7.77
7.75
7.32
7.30
7.26
6.43
6.41
6.21
6.19
6.18
4.42
4.34
4.33
4.31
3.57
3.56
2.73
1.97
1.84
1.79
1.30
1.00
1.01
1.02
1.01
1.03
1.01
1.02
0.97
3.85
2.02
6.13
1.96
3.08
3.11
3.44
6.13
f1 (ppm)

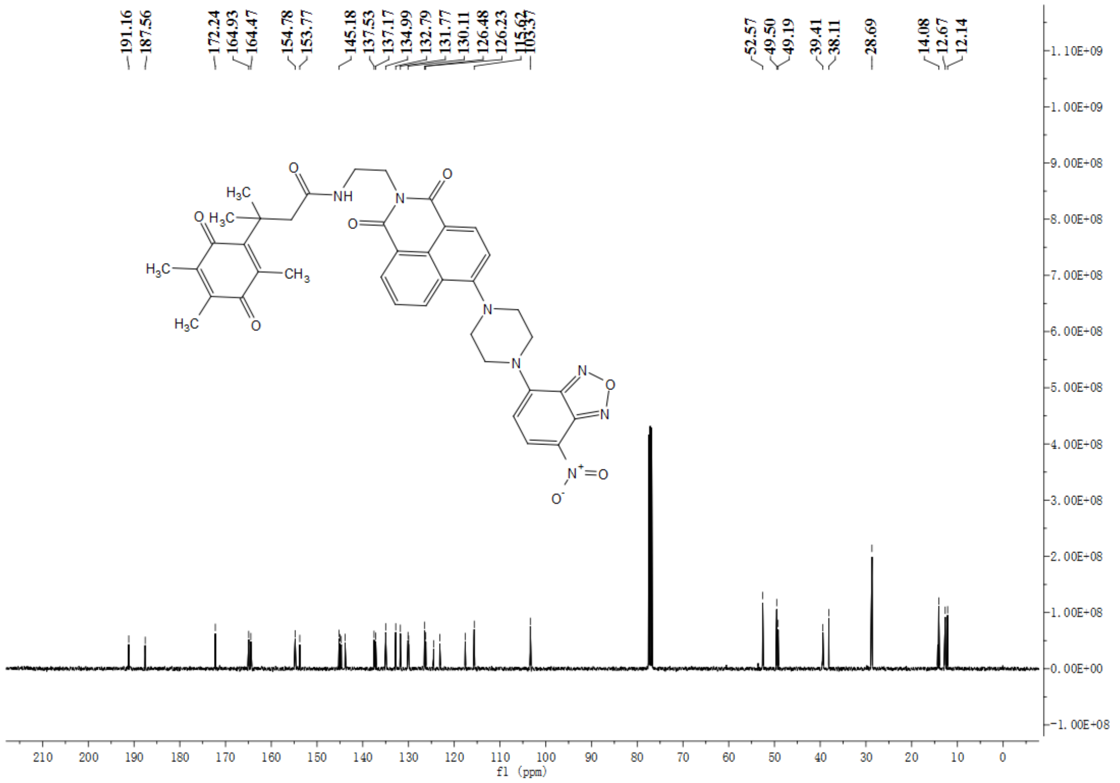

HRMS Spectra

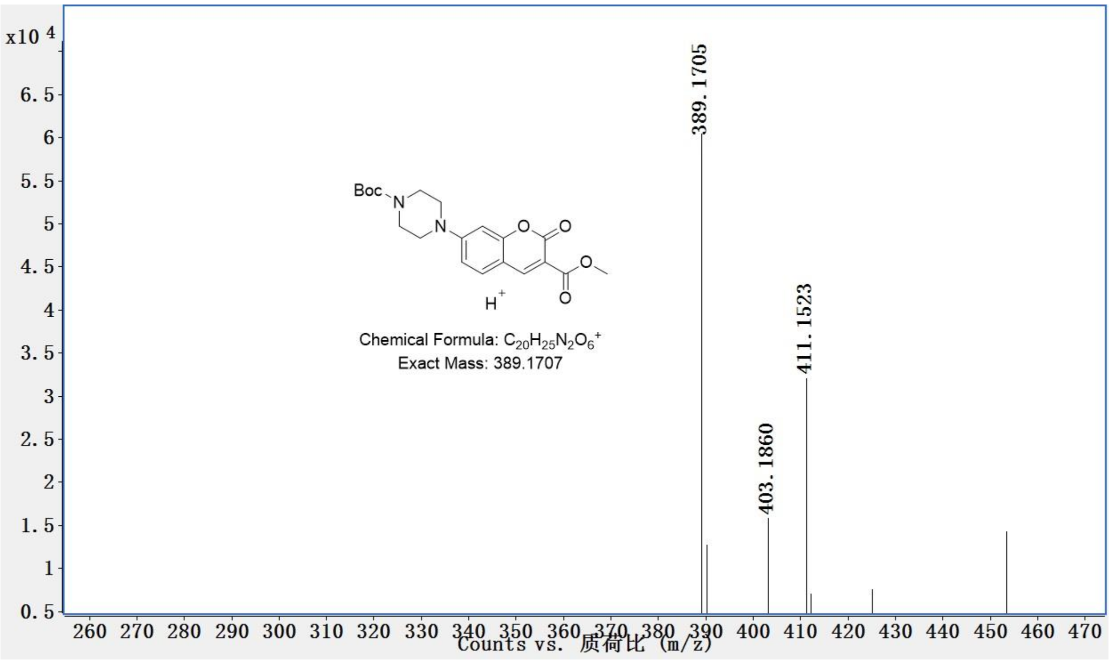

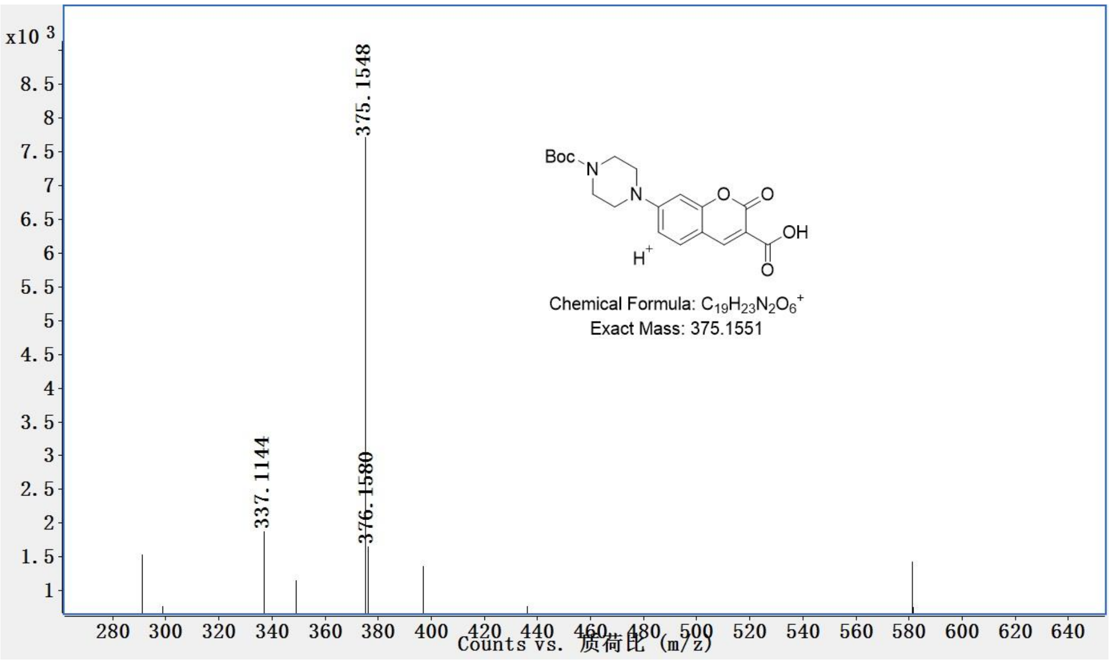

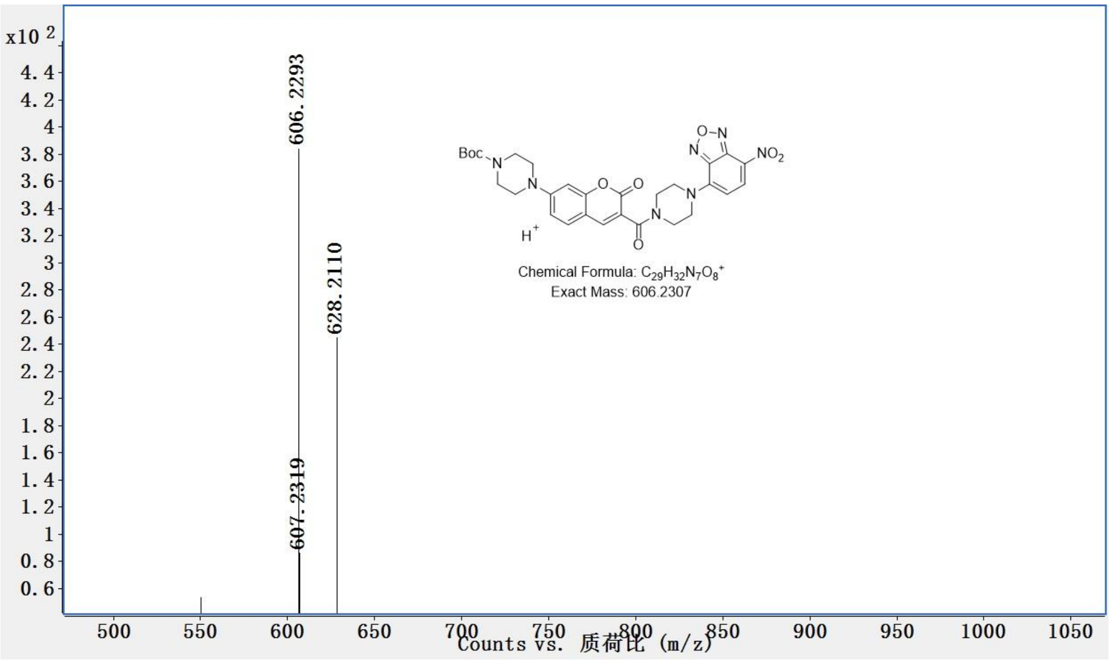

x10 2
606.2293
628.2110
607.2319
Chemical Formula: C29H32N7O8+
Exact Mass: 606.2307
Boc
H+
NO2
Counts vs. 质荷比 (m/z)

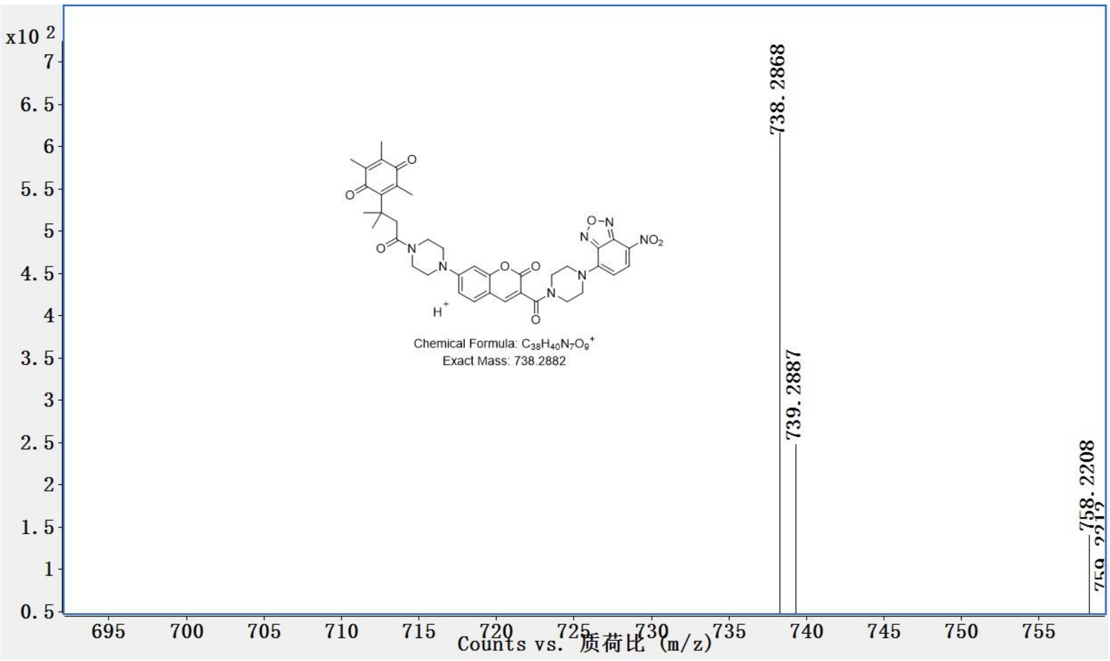

x10 2
738.2868
739.2887
758.2208
Chemical Formula: C38H40N7O9+
Exact Mass: 738.2882
H+
NO2
Counts vs. 质荷比 (m/z)

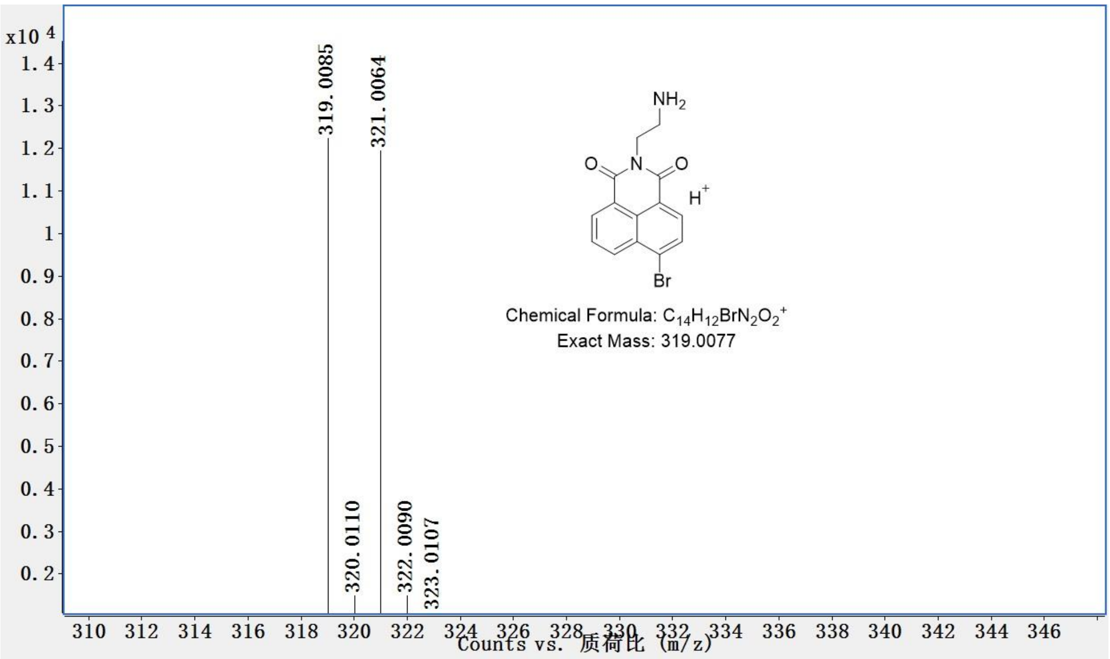

x10 4
1.4
1.3
1.2
1.1
1
0.9
0.8
0.7
0.6
0.5
0.4
0.3
0.2
319.0085
321.0064
320.0110
322.0090
323.0107
NH2
O
N
O
H+
Br
Chemical Formula: C14H12BrN2O2+
Exact Mass: 319.0077
310 312 314 316 318 320 322 324 326 328 330 332 334 336 338 340 342 344 346
Counts vs. 质荷比 (m/z)

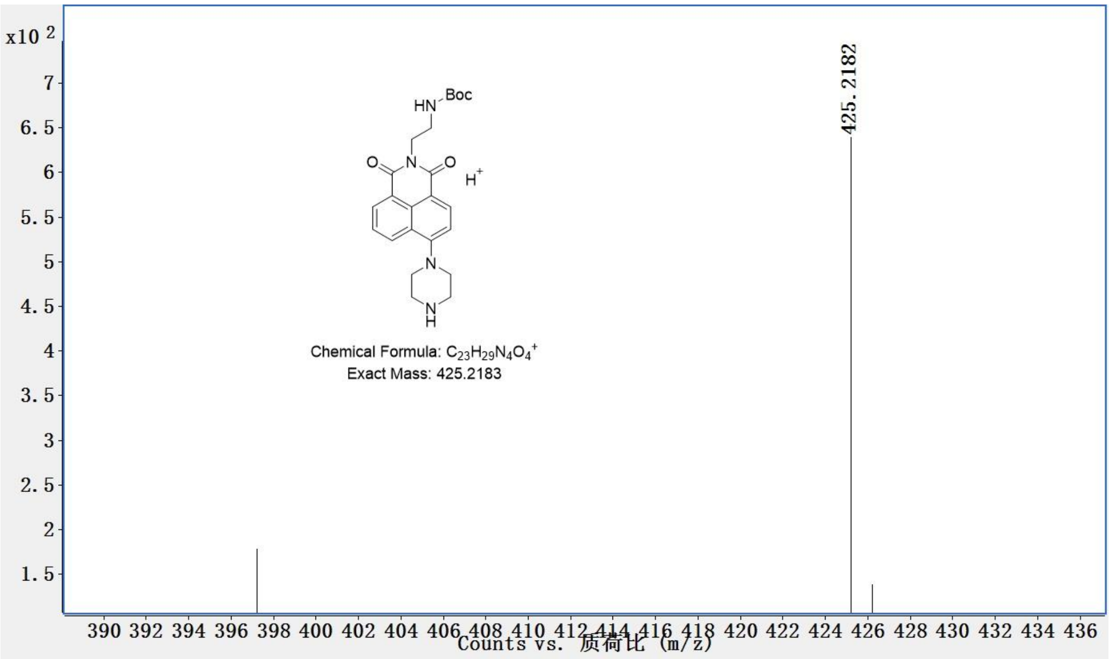

x10 2
7
6.5
6
5.5
5
4.5
4
3.5
3
2.5
2
1.5
425.2182
HN
Boc
O
N
O
H+
N
N
H
Chemical Formula: C23H29N4O4+
Exact Mass: 425.2183
390 392 394 396 398 400 402 404 406 408 410 412 414 416 418 420 422 424 426 428 430 432 434 436
Counts vs. 质荷比 (m/z)

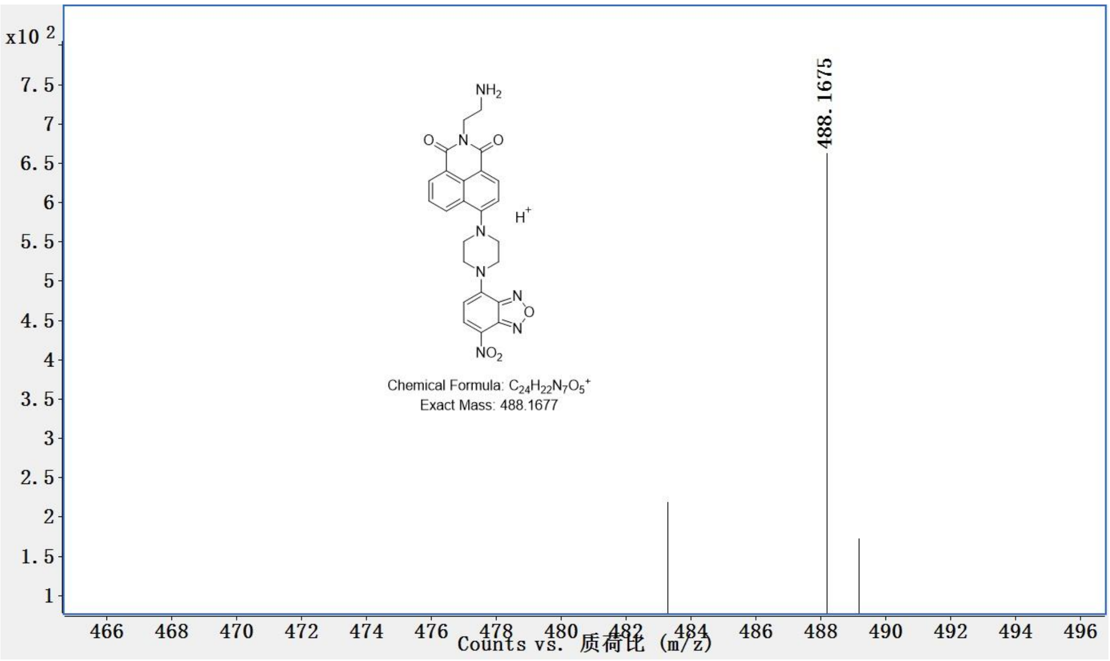
x10 2
488.1675
NH2
H+
NO2
Chemical Formula: C24H22N7O5+
Exact Mass: 488.1677
Counts vs. 质荷比 (m/z)

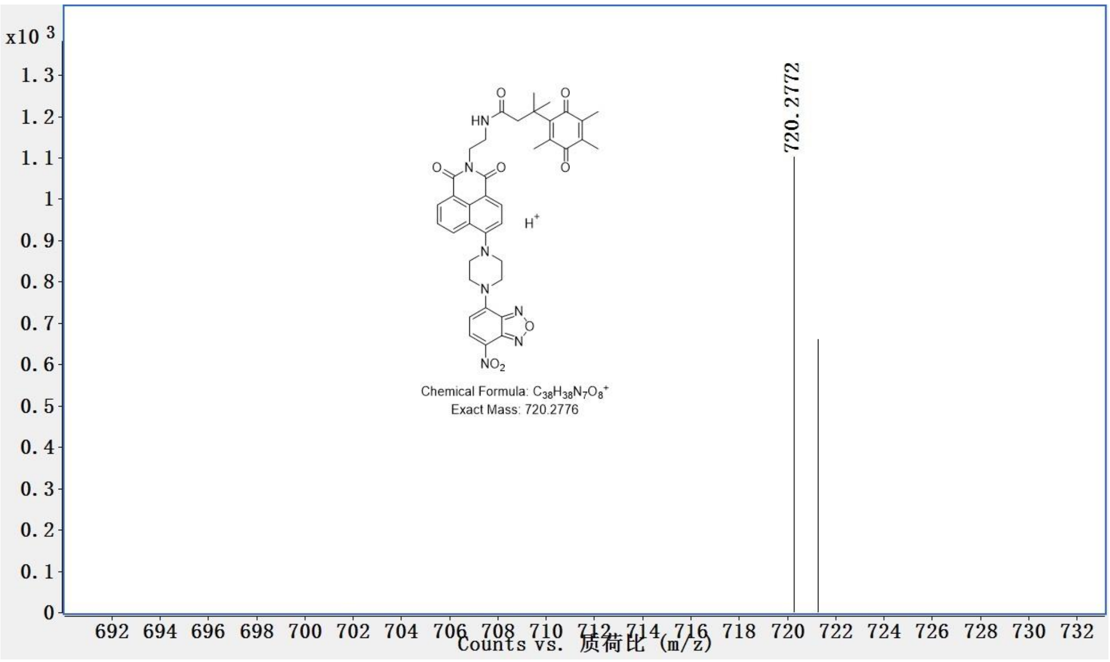
x10 3
720.2772
HN
H+
NO2
Chemical Formula: C38H38N7O8+
Exact Mass: 720.2776
Counts vs. 质荷比 (m/z)
